# Computed tomography-based muscle and fat composition in a Dutch population: a cross-sectional study

**DOI:** 10.1186/s13244-025-02114-2

**Published:** 2025-11-22

**Authors:** Arthur A. R. Sweet, Tim Kobes, Roderick M. Houwert, Saskia Haitjema, Luke P. H. Leenen, Pim Moeskops, Pim A. de Jong, Mark C. P. M. van Baal, Wouter B. Veldhuis

**Affiliations:** 1 Department of Surgery, University Medical Center Utrecht, Utrecht, The Netherlands; 2 Department of Radiology, University Medical Center Utrecht, Utrecht, The Netherlands; 3 Central Diagnostic Laboratory, University Medical Center Utrecht, Utrecht, The Netherlands; 4 Quantib, Rotterdam, The Netherlands

**Keywords:** Body composition, CT, Reference values, Artificial intelligence

## Abstract

**Background:**

Normative adult body composition values from North American patients were recently provided, yet sex- and age-specific reference values for Europeans remain unexplored.

**Materials and methods:**

This cross-sectional study was performed on adult trauma patients who underwent CT imaging that included the abdomen in the University Medical Center Utrecht, a level-1 trauma center, between January 2017 and December 2020. An artificial intelligence algorithm was used to automatically segment muscle and fat components on axial CT images at the level of the third lumbar vertebra. Measurements included areas and attenuation values for muscle and fat, including total muscle areas and “pure-muscle” sub-areas. Skeletal muscle indices were calculated by dividing skeletal muscle areas by the squared height of patients. Age- and sex-specific percentile curves for all parameters were generated.

**Results:**

Of the 2383 adult trauma patients who underwent CT imaging that included the abdomen, 2286 were included. The median age was 53 years (IQR 32–69), and 67.2% were male. The mean BMI was 25.4 ± 4.4 kg/m^2^. The total muscle index decreased with age starting around 60 years in males. In females, the total muscle index decreased with age when intramuscular fat was excluded from the analysis. Mean muscle attenuation of all included muscles showed substantial declines with age in both sexes. Visceral fat areas increased rapidly with age in both sexes, yet with higher values among males.

**Conclusion:**

This study provides CT-derived populational body composition parameters established in Dutch adults, intended to be used as reference values in clinical practice.

**Critical relevance statement:**

Obesity and sarcopenia are risk factors for various diseases and mortality. CT-based body composition assessment provides precise muscle and fat data, enabling personalized treatment plans and early identification of individual risks, ultimately improving patient outcomes and care management strategies.

**Key Points:**

- European reference values of CT-derived muscle and fat parameters are not yet well established.
- This study provides Dutch reference curves and values of CT-derived body composition.
- CT-derived body composition assessment enables personalized risk assessment and treatment.

**Graphical Abstract:**

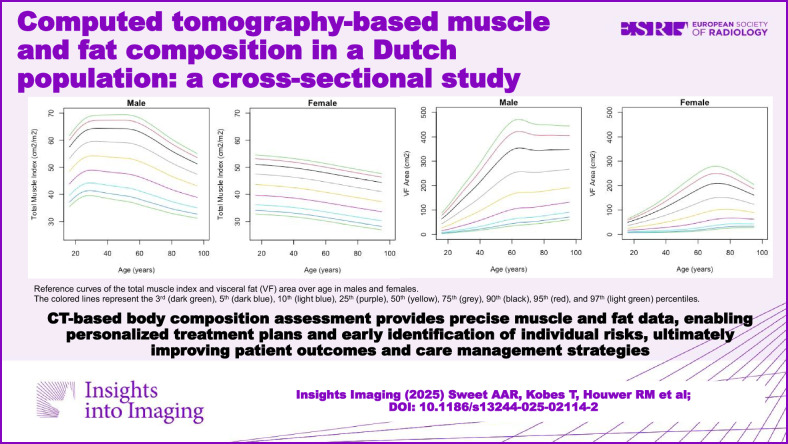

## Introduction

Body composition is a thoroughly investigated topic in health care and public health research because of its association with an increased risk of chronic diseases [1, 2]. Excessive fat mass (i.e., obesity), skeletal muscle mass deficiency (i.e., sarcopenia), and both of these conditions together (i.e., sarcopenic obesity) are risk factors for multiple diseases, drug toxicity, and adverse outcomes [3–5].

Obesity, which is most frequently defined using the body mass index (BMI), has been associated with an increased risk of all-cause mortality and specifically with cardiovascular diseases, metabolic disorders, and cancer, especially in cases of abdominal fat deposition [2, 6–8]. Despite its widespread use, the BMI can be misleading, as it does not distinguish between muscle and fat mass [9]. Also, BMI cannot differentiate between subcutaneous and visceral fat, while specifically the visceral disposition of body fat has been associated with cardiovascular diseases, metabolic disorders, cancer, and mortality [10]. Therefore, there is a trend towards novel quantitative methods to assess fat mass and define obesity, such as bioelectrical impedance analysis, dual-energy X-ray, magnetic resonance imaging, and computed tomography (CT) [9, 11].

Sarcopenia, defined as a progressive and generalized skeletal muscle disorder associated with an increased likelihood of adverse outcomes, is usually identified using dual-energy X-ray or CT [12, 13]. Sarcopenia has been shown to increase the risk of all-cause mortality, functional disability, metabolic disorders, drug toxicity in oncology, and adverse outcomes, for example, after surgery [5, 14, 15]. Furthermore, myosteatosis, defined as excessive fat infiltration in the skeletal muscles, has emerged as an additional risk factor for adverse outcomes and mortality [16]. Myosteatosis can also be quantified using CT, as skeletal muscle attenuation values are inversely related to intramuscular fat deposition [17]. Body composition assessment using CT imaging, often performed around the axial level of the third lumbar vertebra, is currently one of the most accurate methods [12, 13]. This technique is increasingly used in oncology and beyond, as CT images are often already acquired for other purposes in these populations.

A smaller study performed in Dutch healthy kidney donors reported sex-specific percentiles of CT-derived muscle areas and attenuation values, but no age-specific values were given, and fat areas were not investigated [18]. More recently, reference values of adult body composition parameters from an American population were published, yet European reference values specified for age remain unexplored [19]. This study aims to establish normative reference values of adult body composition parameters on CT imaging.

## Materials and methods

### Study design and participants

Our institutional review board approved a waiver of consent (reference number 20-645).

This cross-sectional study was performed on trauma patients admitted to the emergency department of the University Medical Center Utrecht, a level-1 trauma center, between January 2017 and December 2020. This study used data from trauma patients, assuming that they provide a fair representation of the ambulant general population. Eligible patients were identified from the local trauma registry using procedural codes and included if the patients were aged 16 years or older and had a CT that included the abdomen within two days of admission to the emergency department. Patients were excluded if their CT images were not assessable due to severe injury, anatomical deformity, or severe beam hardening artifacts from adjacent osteosynthetic material. This study was conducted in accordance with the eighth revision of the Declaration of Helsinki and was written in adherence to the STROBE (Strengthening the Reporting of Observational Studies in Epidemiology) statement [20].

### CT acquisition and evaluation

CT examinations were obtained with 16 × 0.75 mm collimation (Mx8000 IDT 16), 64 × 0.625 mm collimation (Brilliance 64, iQon Spectral), or 128 × 0.625 mm collimation (Brilliance iCT); all from Philips Medical Systems. Scans were primarily acquired at 120 kilovoltage peak (kVp) and in 3% of the patients at 100 or 140 kVp, with a range of 28 to 272 milliamperes per second selected at the technologist’s discretion, depending on body size.

**Fig. 1:**
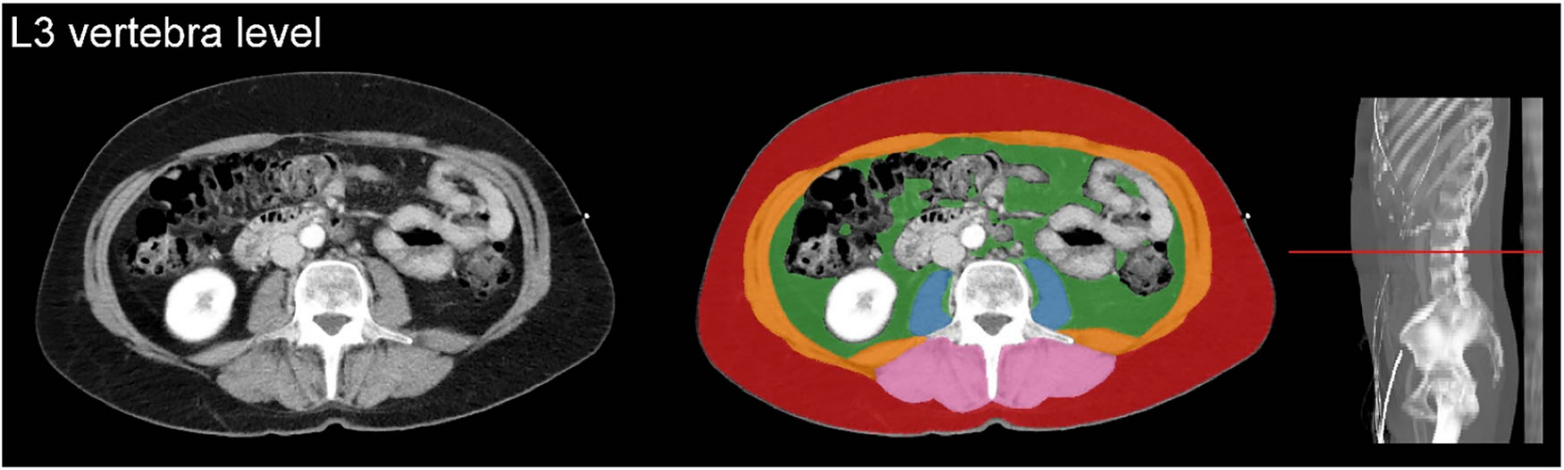
Artificial intelligence-driven segmentation of body composition on axial computed tomography images. Visceral fat (green), subcutaneous fat (red), psoas muscles (blue), abdominal wall muscles (orange), paraspinal muscles (pink)

According to our trauma protocol, all trauma patients who underwent CT immediately after admission (95.6%) received 2 mL/kg contrast medium using the split-bolus technique to assess the portal and arterial phase simultaneously. Axial images at the third lumbar vertebra (L3) level with a slice thickness of 0.9–5 mm were reconstructed and displayed with the soft-tissue setting (window level: 30, window width: 400). Cross-sectional areas of muscles around the level of L3 have been shown to correlate well with whole-body muscle and fat mass [21, 22]. The L3 level was automatically identified, and five axial slices at this level were automatically segmented by an artificial intelligence algorithm (Quantib Body Composition version 0.2.1, Quantib) (Fig. 1) [23]. This AI-driven segmentation method was also described in previous studies and showed high Dice coefficients compared to manual segmentation [24–26]. All segmented images were manually screened for errors (e.g., another level than L3 or incorrect segmentation) by A.S. under the supervision of a board-certified abdominal radiologist (W.V.), and corrected if needed using the Medical Imaging Interaction Toolkit, a free, open-source software system [27]. Presented tissue-area and attenuation parameters were primarily derived from the complete anatomic areas as directly segmented by the algorithm. Secondarily, tissue-area and attenuation parameters were derived from sub-areas within the segmented area with an attenuation value above −30 Hounsfield units (HU), a cut-off point that separates macroscopic intramuscular fat (< −30 HU) from muscular tissue (≥ −30 HU) in the segmented muscle compartment. We will refer to this sub-area as the “pure-muscle area”.

### Explanatory and response variables

Demographics (i.e., age and sex), American Society of Anesthesiologists (ASA) classification, weight, height, and CT images were retrieved from the medical records. The following body composition parameters were automatically retrieved from the axial CT images: psoas muscle area, abdominal wall muscle area (rectus abdominis, transverse, internal and external oblique muscles, quadratus lumborum), paraspinal muscle area (erector spinae and multifidus), subcutaneous fat area, and visceral fat area. Also, attenuation values in HU of all specific muscle areas and fat areas were measured and presented per muscle, as it was previously shown that attenuation values vary between muscles [28]. To adjust for the patients’ height in assessing the muscle areas, skeletal muscle indices of all specific muscle areas and the total muscle area were calculated by dividing the muscle area by the squared height of the patient (cm^2^/m^2^).

### Statistical analysis

Baseline characteristics were presented as mean ± standard deviation (SD) for parametric continuous variables, median with interquartile range (IQR) for non-parametric continuous variables, and absolute numbers with percentages for categorical variables. Quantile-Quantile plots were used to assess the variables for normality. Missing data were assumed missing completely at random, and no imputation techniques were used to establish the reference values. Statistical analyses were performed using Stata 13.0 (StataCorp LP). Additional analyses to create reference curves with percentiles and the tables with corresponding values for each percentile were performed using RStudio 1.4.1717 for Mac using the *gamlss* package. The reference curves display the following percentiles: p3, p5, p10, p25, p50, p75, p90, p95, p97. Additional testing showed that all muscle variables were normally distributed, and all fat variables were non-normally distributed. The appropriate distributions, normal for all muscle-related variables and gamma for all adipose tissue-related variables, were used in the analysis to create the reference curves. A chi-squared test was used to test for differences in proportions of moderate and severe overweight in our study population versus the Dutch national population. A *p*-value of < 0.05 was considered statistically significant.

## Results

A total of 2383 adult trauma patients underwent CT imaging that included the abdomen within two days from the date of admission to the emergency department during the study period. After excluding 97 patients with CT images that were not assessable due to severe injury, anatomical deformity, or severe beam hardening artifacts from adjacent osteosynthesis material, 2286 patients were included for analysis. The median age was 53 years (IQR 32–69), and 67.2% were male (Table 1). The majority (80.4%) had an ASA of 1–2 before the trauma, while 19.7% had an ASA of 3–4. The mean BMI was 25.4 ± 4.4 kg/m^2^.

**Table 1:** Cohort characteristics

Variable	Total cohort	Missings
	*n* = 2286	%
Age, median years (IQR)	53 (32–69)	0.0
Male, *n* (%)	1536 (67.2)	0.0
ASA, *n* (%)		5.7
1	950 (44.1)	
2	783 (36.3)	
3	398 (18.5)	
4	25 (1.2)	
BMI, mean kg/m^2^ ± SD	25.4 ± 4.4	28.9
Weight, mean kg ± SD	79.0 ± 16.0	26.1
Height, mean m ± SD	176.4 ± 10.1	22.7

*IQR* interquartile range, *SD* standard deviation, *ASA* American Association of Anesthesiologists, *BMI* body mass index, *kg* kilogram, *m* meter

### Skeletal muscle indices and mean attenuation

**Fig. 2:**
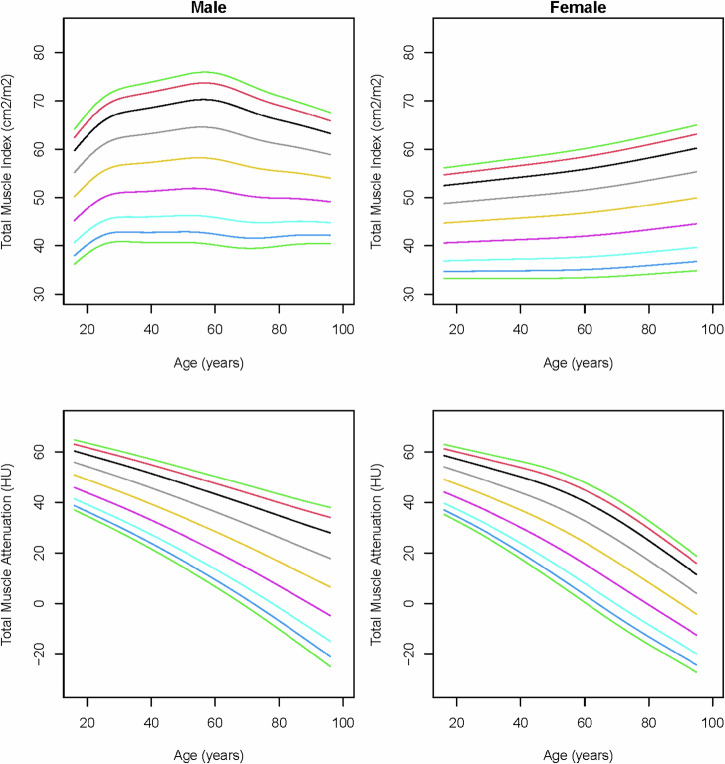
Reference curves of total muscle index and total muscle attenuation (Hounsfield units) of the complete areas over age in males and females. The colored lines represent the 3rd (dark green), 5th (dark blue), 10th (light blue), 25th (purple), 50th (yellow), 75th (gray), 90th (black), 95th (red), and 97th (light green) percentiles

In males, the total muscle index showed an increasing trend starting in young adults, followed by a continuous decrease, beginning around 60 years of age (Fig. 2). In females, the total muscle index showed a slight increase with age, predominantly caused by the increasing abdominal wall and paraspinal muscle indices (Fig. 2; Supplemental Figs. 5 and 7). The total muscle attenuation showed substantial declines with age in both sexes (Fig. 2).

**Fig. 3:**
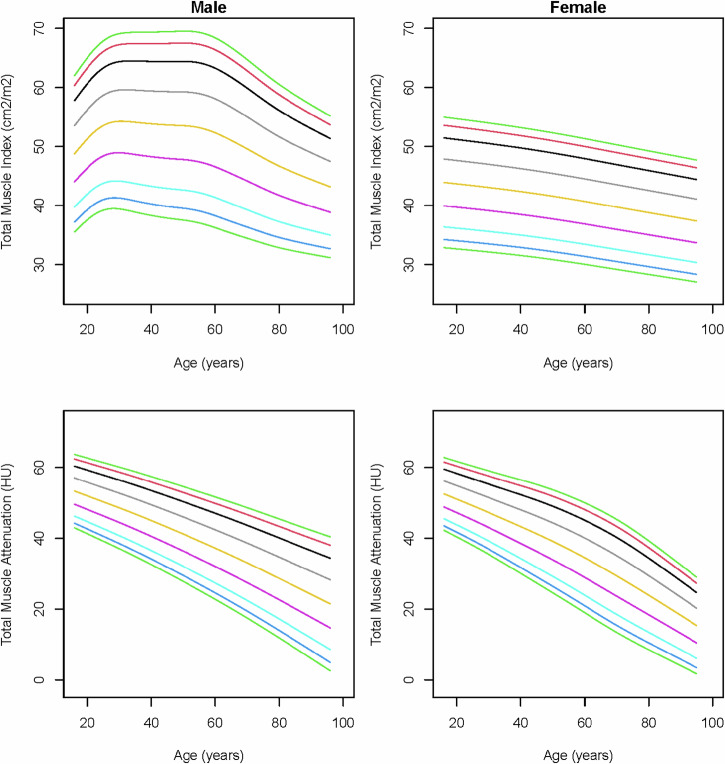
Reference curves of total muscle index and total muscle attenuation (Hounsfield units) of the pure-muscle sub-areas over age in males and females. The colored lines represent the 3rd (dark green), 5th (dark blue), 10th (light blue), 25th (purple), 50th (yellow), 75th (gray), 90th (black), 95th (red), and 97th (light green) percentiles

When deriving the total muscle index from the pure-muscle sub-areas with HU ≥ −30, the paraspinal and abdominal wall muscle indices, and thereby the total muscle index, showed steeper decreases with age in males (Fig. 3; Supplemental Figs. 6 and 8). The pure-muscle index showed that, also in females, the total volume of pure-muscle decreases with age, where the muscle index based on the complete area shows that the total muscle volume slightly increases with age, due to an increase in macroscopic intramuscular fat that is larger than the decrease in pure-muscle.

### Fat areas and mean attenuation

**Fig. 4:**
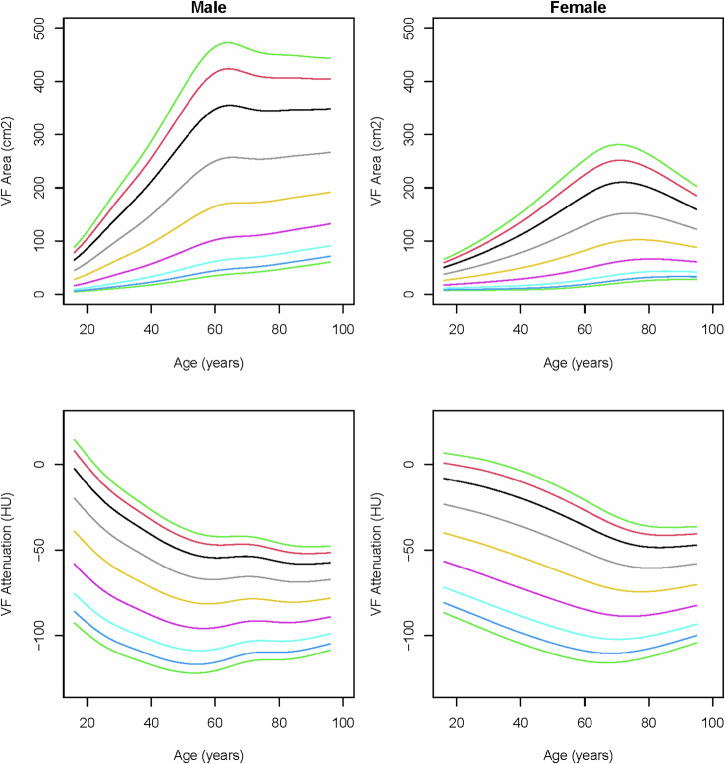
Reference curves of visceral fat (VF) area and visceral fat attenuation (Hounsfield units) over age in males and females. The colored lines represent the 3rd (dark green), 5th (dark blue), 10th (light blue), 25th (purple), 50th (yellow), 75th (gray), 90th (black), 95th (red), and 97th (light green) percentiles

In males, visceral fat areas increased rapidly with age until around the age of 65, after which the increase levels out (Fig. 4). A similar dynamic was seen in females, but with lower peak values and a decreasing trend starting around 75 years. In both sexes, visceral fat attenuation decreased from young adulthood until around 60 years, and slowly increased in older ages.

**Fig. 5:**
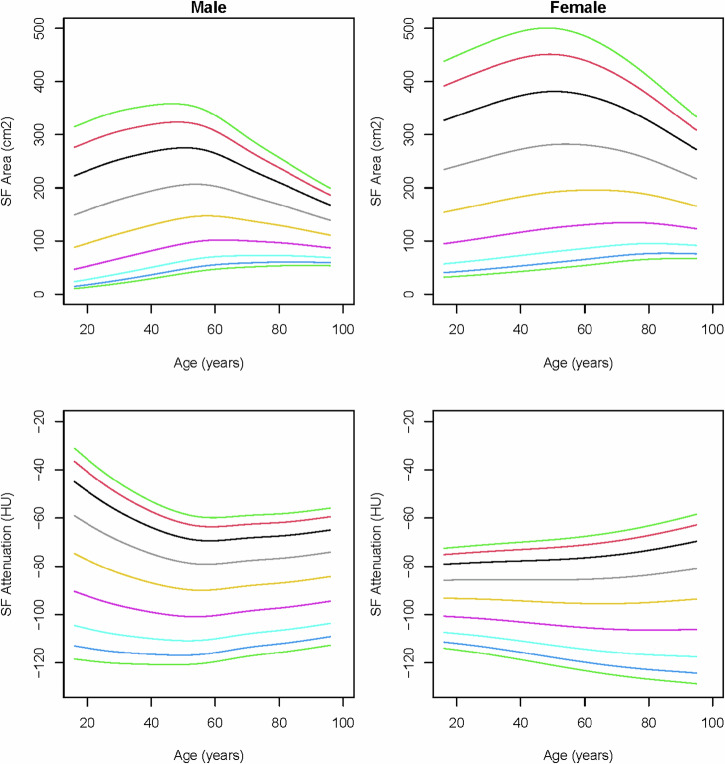
Reference curves of subcutaneous fat (SF) area and subcutaneous fat attenuation (Hounsfield units) over age in males and females. The colored lines represent the 3rd (dark green), 5th (dark blue), 10th (light blue), 25th (purple), 50th (yellow), 75th (gray), 90th (black), 95th (red), and 97th (light green) percentiles

Subcutaneous fat areas were highest among middle aged individuals in both sexes and showed higher values in females than males. In males, subcutaneous fat attenuation values decreased with age, followed by a slight increase around the age of 60 years (Fig. 5). Besides a slight decrease in young females, subcutaneous fat attenuation remains fairly unaffected by age.

The exact percentile values of all body composition parameters were given in the supplementary tables and figures. Also, the reference values of total fat area, muscle-to-fat ratio, and visceral-to-subcutaneous fat ratio were provided in the supplements.

## Discussion

This study established CT-derived body composition reference values in Dutch adults and provided insight into the body composition of males and females across the adult lifespan. To our knowledge, this is the first European study to provide such reference values. Knowledge of normative ranges can guide physicians in identifying patients with excessive fat mass or muscle deficiencies, which both pose risks of various diseases, adverse outcomes after disease, drug toxicity, and mortality. These reference curves can be used in clinical practice to identify patients at risk for complications such as pneumonia or delirium or mortality.

The trends of male muscle indices calculated using the complete muscle areas were comparable to the pure-muscle areas without macroscopic fat tissue (i.e., areas with attenuation ≥ −30 HU), although the latter muscle indices were generally lower. In females, the total muscle indices increased slightly with age, predominantly caused by the increasing intramuscular fat depositions of the paraspinal muscles and, to a lesser extent, the abdominal muscles. When assessing the pure-muscle areas, all female muscle indices showed declines with age. The present study results were compared to American body composition values that were based on specified HU ranges for muscle and fat, whereas our segmentation was based on body anatomy [19]. To provide an appropriate comparison, the American values of white non-Hispanic patients were compared to Dutch muscle indices based on pure-muscle areas. The total muscle indices in Dutch males and females followed a descending trend with age, similar to the American reference values [19]. Furthermore, similar trends were seen in visceral and subcutaneous fat increases with age in Dutch and American patients, yet with higher values of the upper percentiles in American patients [19]. No comparison of normative age-specific muscle and fat attenuation values could be made, as no such values have been published yet for the American population.

Various CT-derived fat and muscle mass thresholds have been established based on associations with disease or adverse outcomes after disease. Thresholds of visceral fat areas, to predict an increased risk of cardiovascular or metabolic disease, were reported between 124–142 cm^2^ in males and between 91.1−173 cm^2^ in females [29–33]. Applying these thresholds to the full age range of our data, the majority of males of 50 years or older and the majority of females of 70 years or older would be at increased risk of cardiovascular and metabolic disease. However, our reference curves show a “physiological” increase of visceral fat mass with age in both sexes, and it may therefore be necessary to also account for age when assessing the risk of certain diseases. For instance, in post-menopausal females, 100 cm^2^ visceral fat area is a median value, but this same value is even higher than the 97th percentile in young females. Reasonably, the risks attached to this same amount of fat mass differ between the young and the elderly. Previous literature in healthy and oncologic populations reported total skeletal muscle index thresholds to define sarcopenia varying between 41.6−52.4 cm^2^/m^2^ in males and 32.0–39 cm^2^/m^2^ in females [18, 34–36]. These thresholds were all established using segmentation methods in which only muscle fibers without macroscopic intramuscular fat were included (i.e., attenuation ≥ −30 HU). When applying these thresholds to our values of pure-muscle indices based on muscle fibers only, more than half of the 60-year-old males and more than half of the 80-year-old females may be classified as sarcopenic. However, similar to the increase of fat with age, muscle mass follows a certain “physiological” decrease with age, which is more apparent in males than in females. Knowledge of age- and sex-specific percentiles of fat and muscle mass can be important for accurate personalized risk assessment, as fixed body composition cut-off values may result in an underestimation of the risk in the young and an overestimation in the elderly. The presented data with percentile scores may be preferable to identify visceral obesity and sarcopenia compared to fixed cut-offs, although the (incremental) predictive value for adverse outcomes requires further study.

A systematic review of studies on the effects of myosteatosis in oncologic patients, mostly measured using muscle attenuation, reported significant associations with complications and mortality [16]. However, when identifying patients at risk for disease based on attenuation values, it should be considered that these values could have been affected by other objects or body parts, but also by medical conditions that are less likely to contribute to the risk of disease or mortality. For instance, lumbar back pain and arthrosis of the hip could affect attenuation values, besides known risk factors of a lower muscle density, such as age, sex, and obesity [17]. Similarly, muscle areas of the psoas muscle were described to be affected by spine surgery, lumbar back pain, degenerative instability, vertebral fracture, and deformity [37]. Also, it should be taken into account that other objects or body parts may create artifacts in attenuation values on CT that can introduce bias if not corrected for. For instance, patients are preferably scanned with both arms up in the CT to prevent beam hardening artifacts in the abdominal area, but in cases of inflexibility or severe injuries, one or both arms may be kept down, resulting in artifactually lower attenuation values than in patients scanned with both arms up. This phenomenon applies especially to the psoas muscles, as the beam hardening created by the arms reaches exactly through these muscles in most cases.

Besides the early identification of patients at risk for disease, complications, or mortality, body composition assessment may also be helpful to develop personalized treatment plans, as there is growing evidence that using body composition rather than body surface area for determining chemotherapy doses may reduce toxicity [38]. Similarly, body composition parameters may be used for personalized contrast dosing [24]. When artificial intelligence-derived automatic body composition assessment is fully implemented in standard CT management, the benefits, as mentioned earlier, become available for all patients undergoing abdominal CT without additional costs or loss of time.

This study has its limitations. First, we aimed to provide normative reference values, which could preferably be used in all medical disciplines, but we only used trauma patients from a single center for this purpose. However, in contrast to cardiovascular or oncologic patients, trauma patients are more likely to present with body compositions that are still unaffected by their condition, which in most cases only consists of traumatic injuries. The Dutch national registry reported on percentages of the population above 20 years of age who were moderately overweight (BMI between 25.0 and 30.0) and severely overweight (BMI above 30.0). Although the national proportions (moderate overweight 36.1%; severe overweight 14.7%) appeared comparable to those observed in the study population (moderate overweight 34.4%; severe overweight 12.7%), chi-squared testing showed a significant difference between the groups (*p* < 0.05). It should be noted, however, that unlike the national registry, this study also included patients aged 16 to 20 years. Second, we also established reference values of muscle and fat attenuation values, but as the vast majority of trauma patients were administered a contrast agent, care is needed when extrapolating these values to unenhanced CT. An effect of contrast agent on muscle attenuation was previously shown, but the extent to which it exactly affects muscle attenuation values remains unknown and may vary between muscle areas [39]. Nevertheless, the vast majority of the abdominal CT scans are acquired with intravenous contrast. Furthermore, contrast-induced attenuation changes in fat are already minimal, and with the advent of dual-energy and photon-counting CT acquisition, this potential issue will be further mitigated. Third, data on height were missing in 22.7% of the included patients, resulting in a smaller study population to determine reference values of the muscle indices compared to the fat areas. Last, as race is not documented in Dutch hospitals or in the Dutch national registry, variations in body composition between races described by previous literature have not been taken into account in the present study [40]. While exact percentages remain unknown, it can be stated that the vast majority of the Dutch population is white.

In conclusion, this study provided CT-derived Dutch body composition reference values. Ultimately, patients could benefit from body composition assessment by developing personalized treatment plans or by early identification of their personalized risks of disease, complications, or mortality.

## Supplementary information

ELECTRONIC SUPPLEMENTARY MATERIAL

## Data Availability

Data generated or analyzed during the study are available from the corresponding author upon request.

## Abbreviations

ASA: American Society of Anesthesiologists
BMI: Body mass index
CT: Computed tomography
HU: Hounsfield units
IQR: Interquartile range
SD: Standard deviation
