# Supplementary material for: Computed tomography-based muscle and fat composition in a Dutch population: a cross-sectional study
Source: Insights Imaging. 2025 Nov 22;16:260. doi: 10.1186/s13244-025-02114-2 (PMC12640395; doi:10.1186/s13244-025-02114-2)
Supplement: Supplementary file 1 — ELECTRONIC SUPPLEMENTARY MATERIAL [file 13244_2025_2114_MOESM1_ESM.pdf]

Computed tomography-based muscle and fat composition in a Dutch population: a cross-sectional study

ELECTRONIC SUPPLEMENTARY MATERIAL

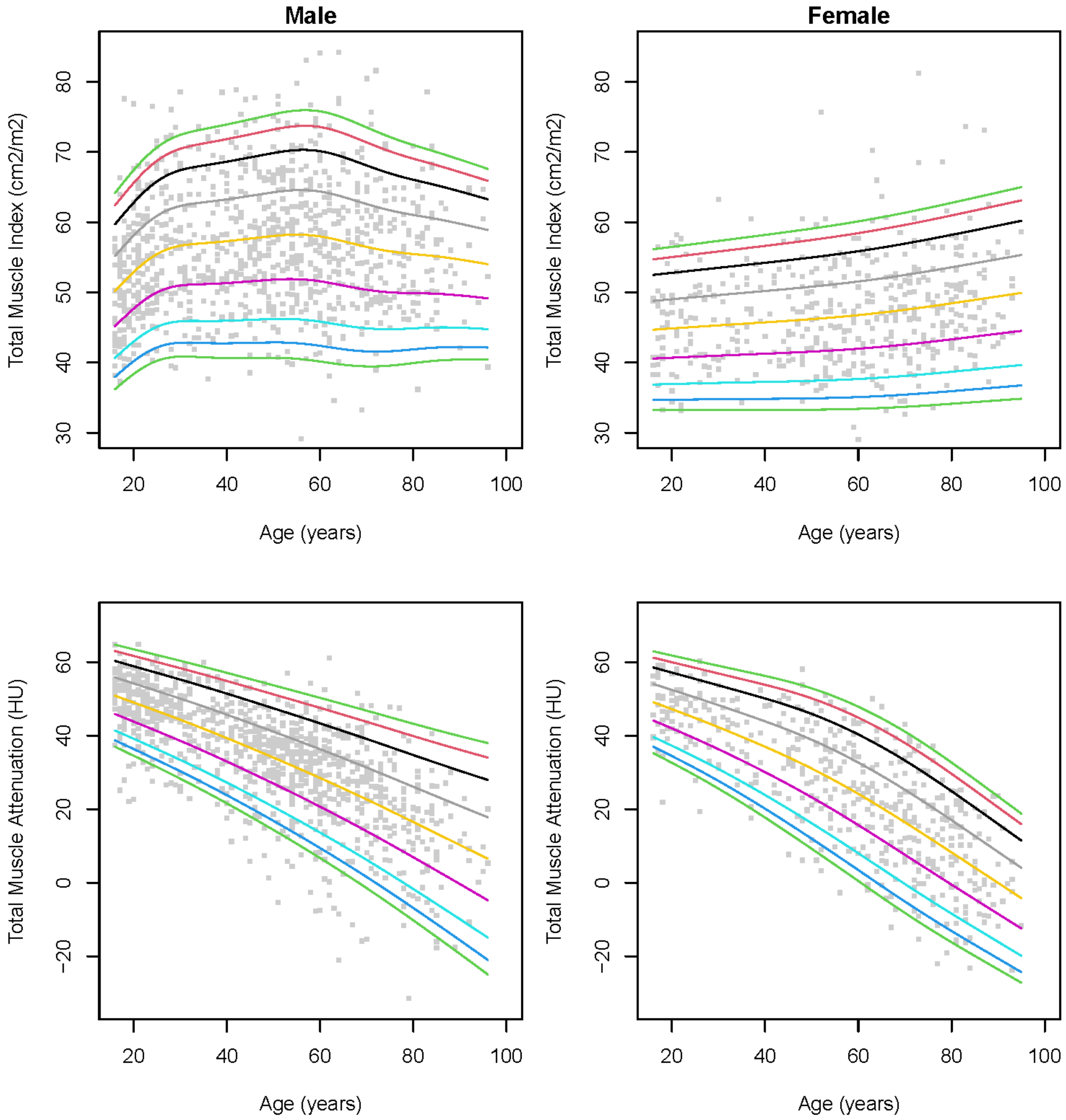

Supplemental figure 1. Reference curves of total muscle index and total muscle attenuation (Hounsfield units) of the complete areas over age in males and females. The colored lines represent the 3rd (dark green), 5th (dark blue), 10th (light blue), 25th (purple), 50th (yellow), 75th (grey), 90th (black), 95th (red), and 97th (light green) percentiles.

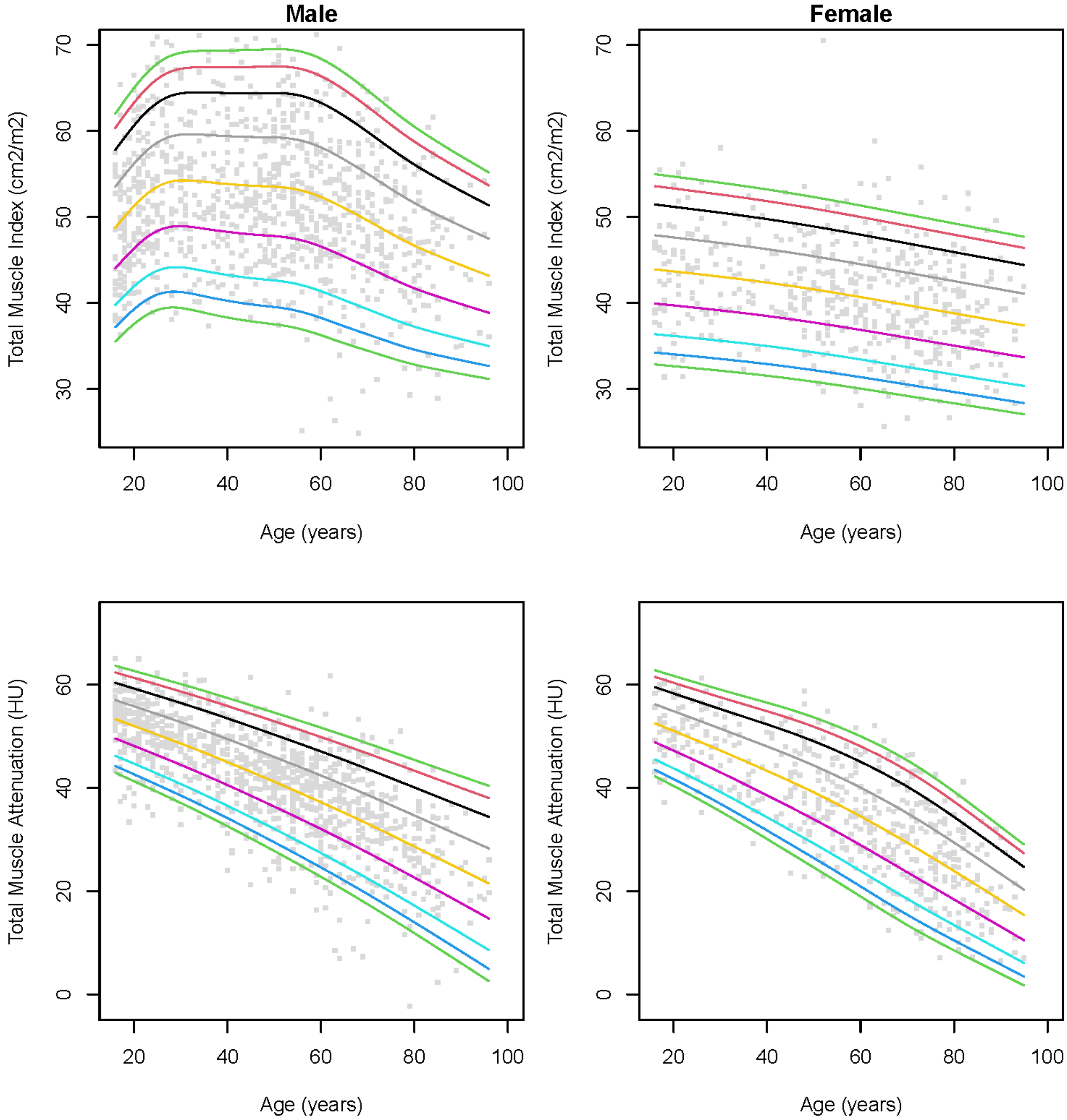

Supplemental figure 2. Reference curves of total muscle index and total muscle attenuation (Hounsfield units) of the pure-muscle sub-areas over age in males and females. The colored lines represent the 3rd (dark green), 5th (dark blue), 10th (light blue), 25th (purple), 50th (yellow), 75th (grey), 90th (black), 95th (red), and 97th (light green) percentiles.

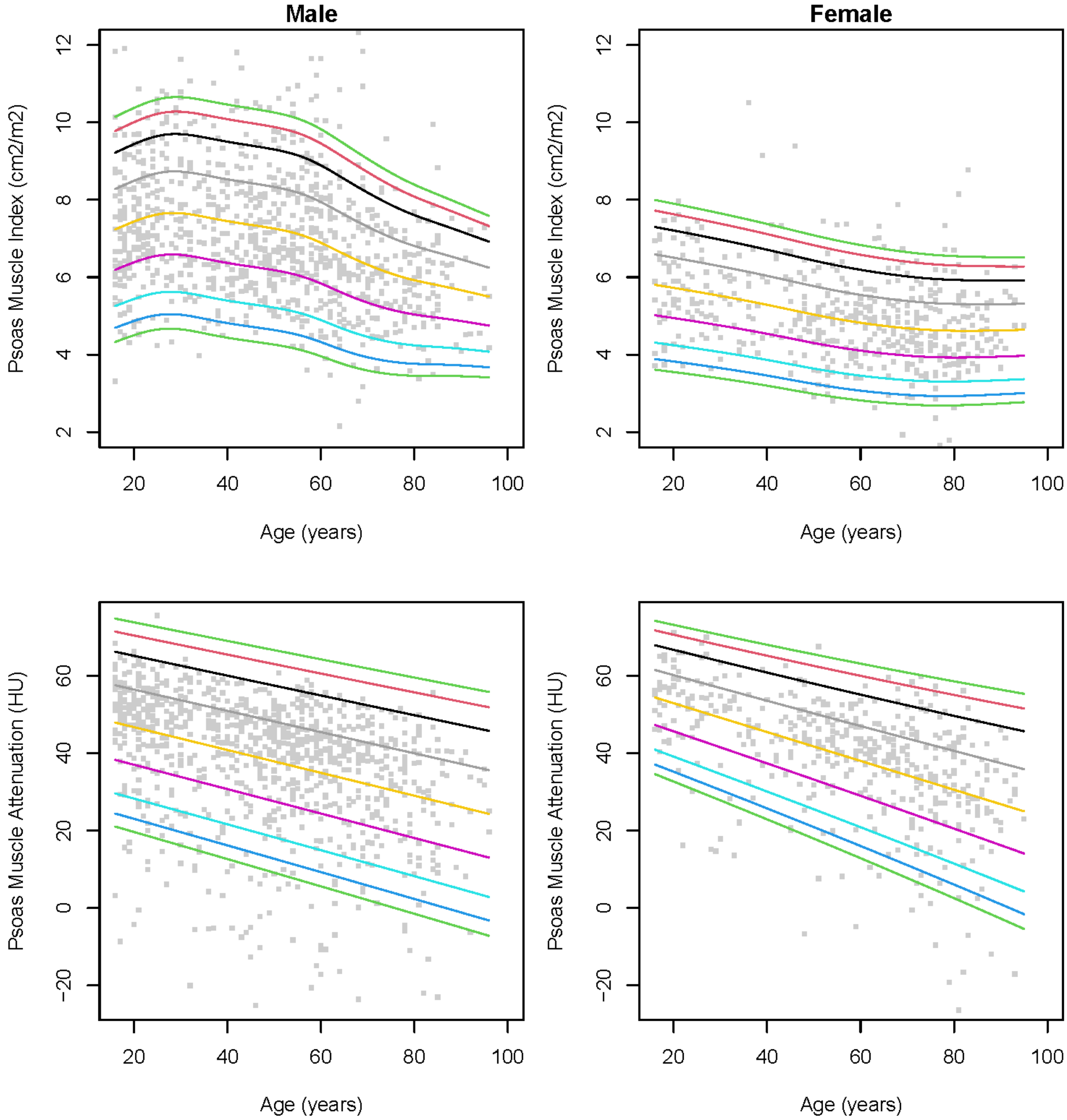

Supplemental figure 3. Reference curves of psoas muscle index and psoas muscle attenuation (Hounsfield units) of the complete areas over age in males and females. The colored lines represent the 3rd (dark green), 5th (dark blue), 10th (light blue), 25th (purple), 50th (yellow), 75th (grey), 90th (black), 95th (red), and 97th (light green) percentiles.

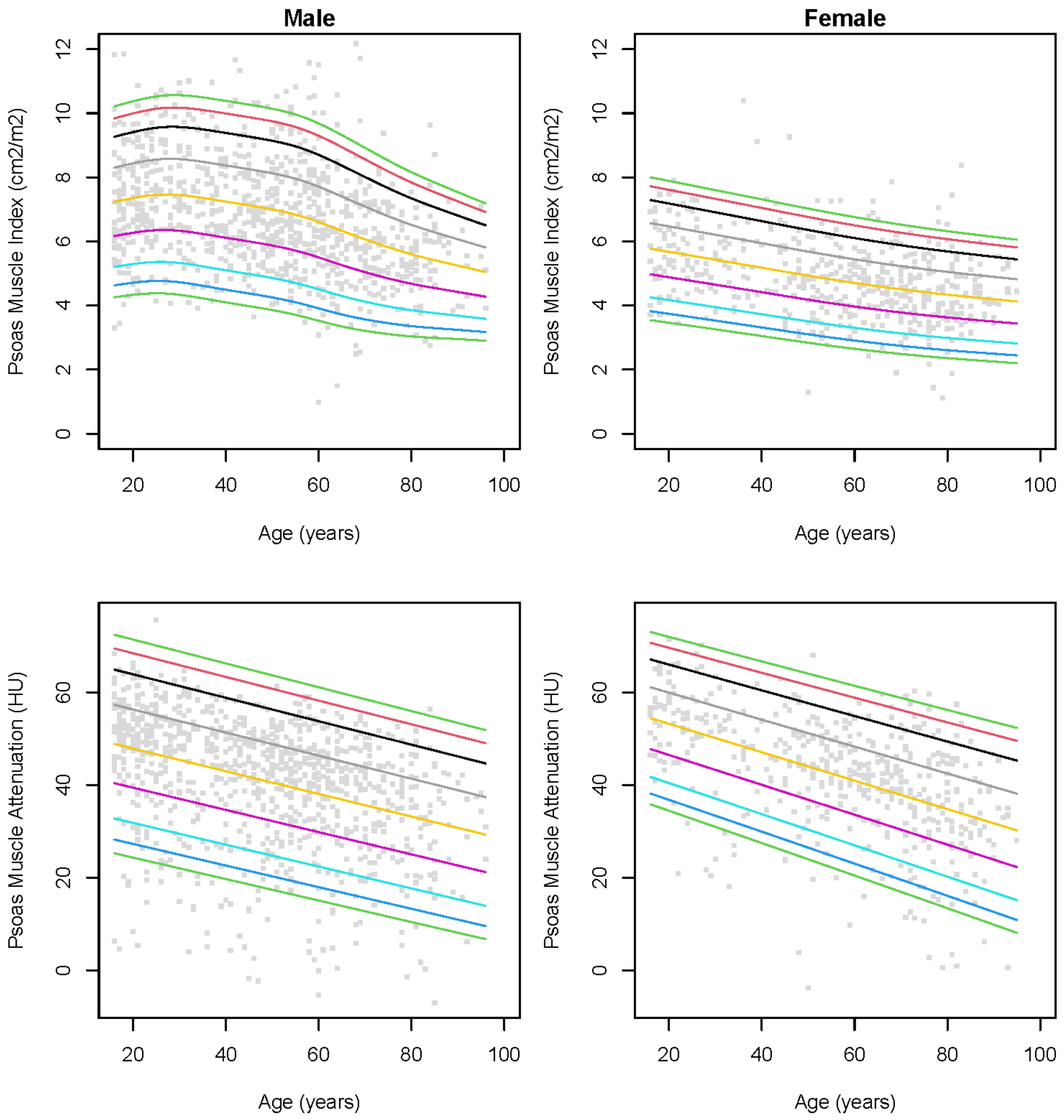

Supplemental figure 4. Reference curves of psoas muscle index and psoas muscle attenuation (Hounsfield units) of the pure-muscle sub-areas over age in males and females. The colored lines represent the 3rd (dark green), 5th (dark blue), 10th (light blue), 25th (purple), 50th (yellow), 75th (grey), 90th (black), 95th (red), and 97th (light green) percentiles.

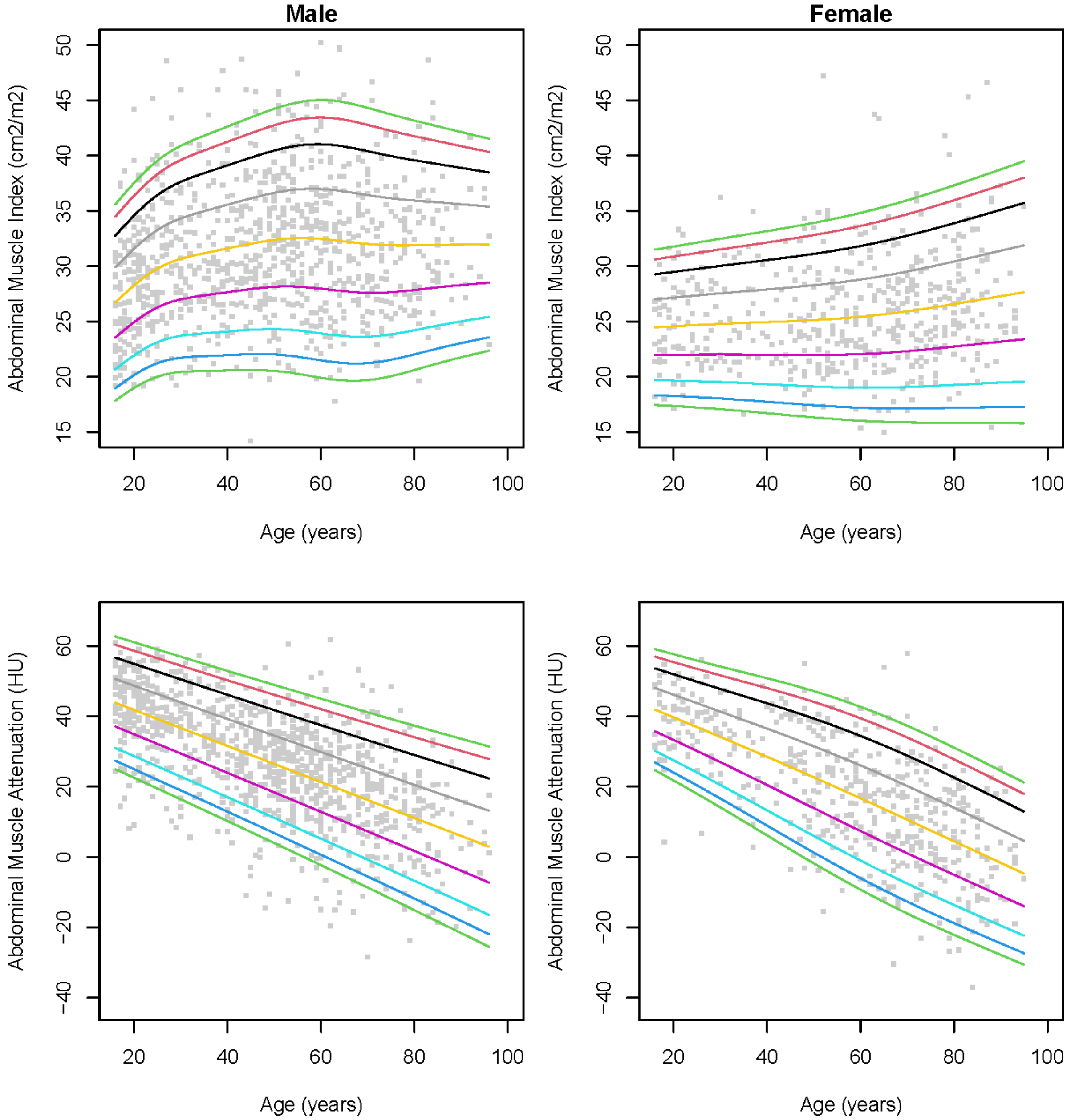

Supplemental figure 5. Reference curves of abdominal muscle index and abdominal muscle attenuation (Hounsfield units) of the complete areas over age in males and females. The colored lines represent the 3rd (dark green), 5th (dark blue), 10th (light blue), 25th (purple), 50th (yellow), 75th (grey), 90th (black), 95th (red), and 97th (light green) percentiles.

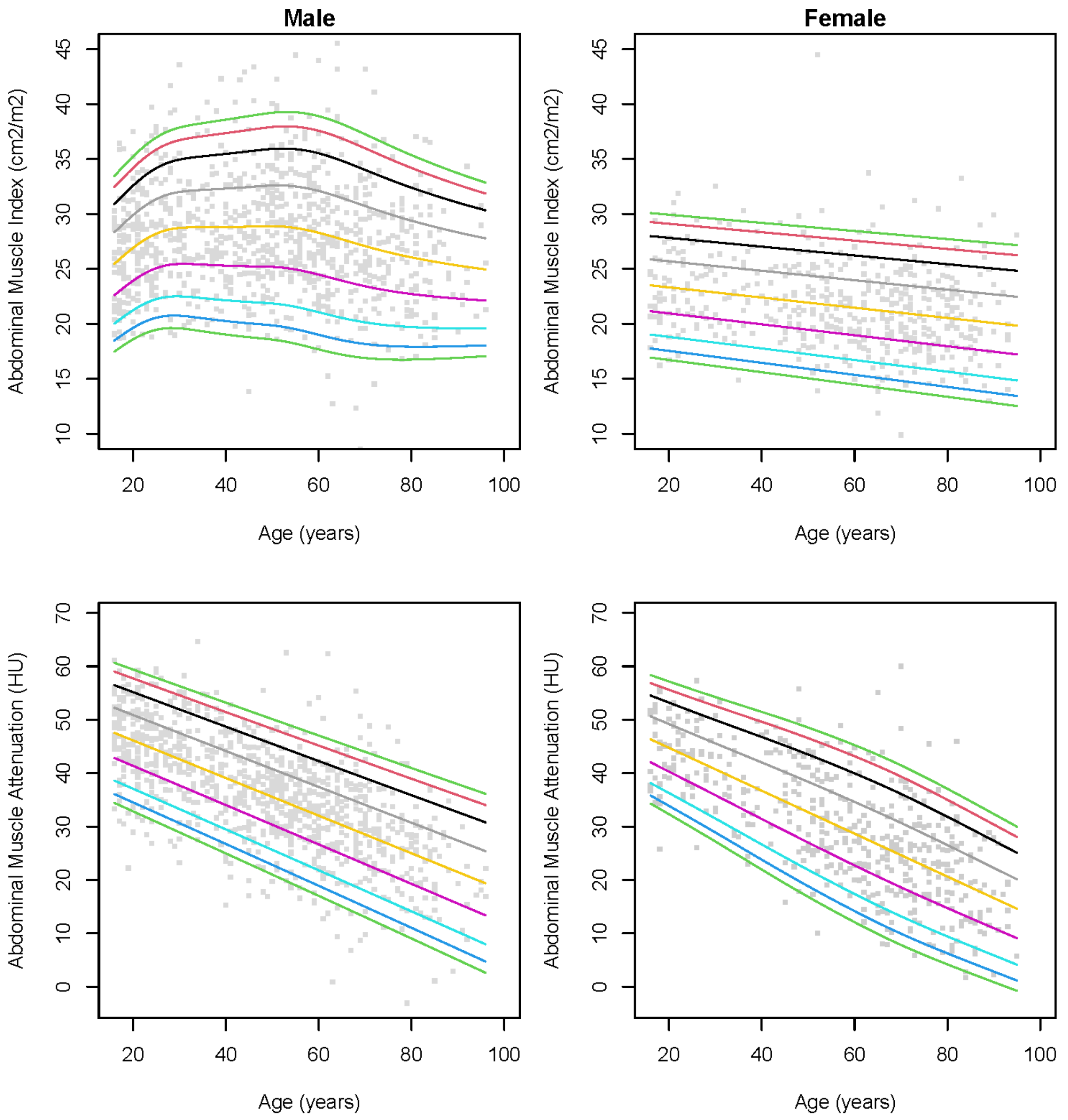

Supplemental figure 6. Reference curves of abdominal muscle index and abdominal muscle attenuation (Hounsfield units) of the pure-muscle sub-areas over age in males and females. The colored lines represent the 3rd (dark green), 5th (dark blue), 10th (light blue), 25th (purple), 50th (yellow), 75th (grey), 90th (black), 95th (red), and 97th (light green) percentiles.

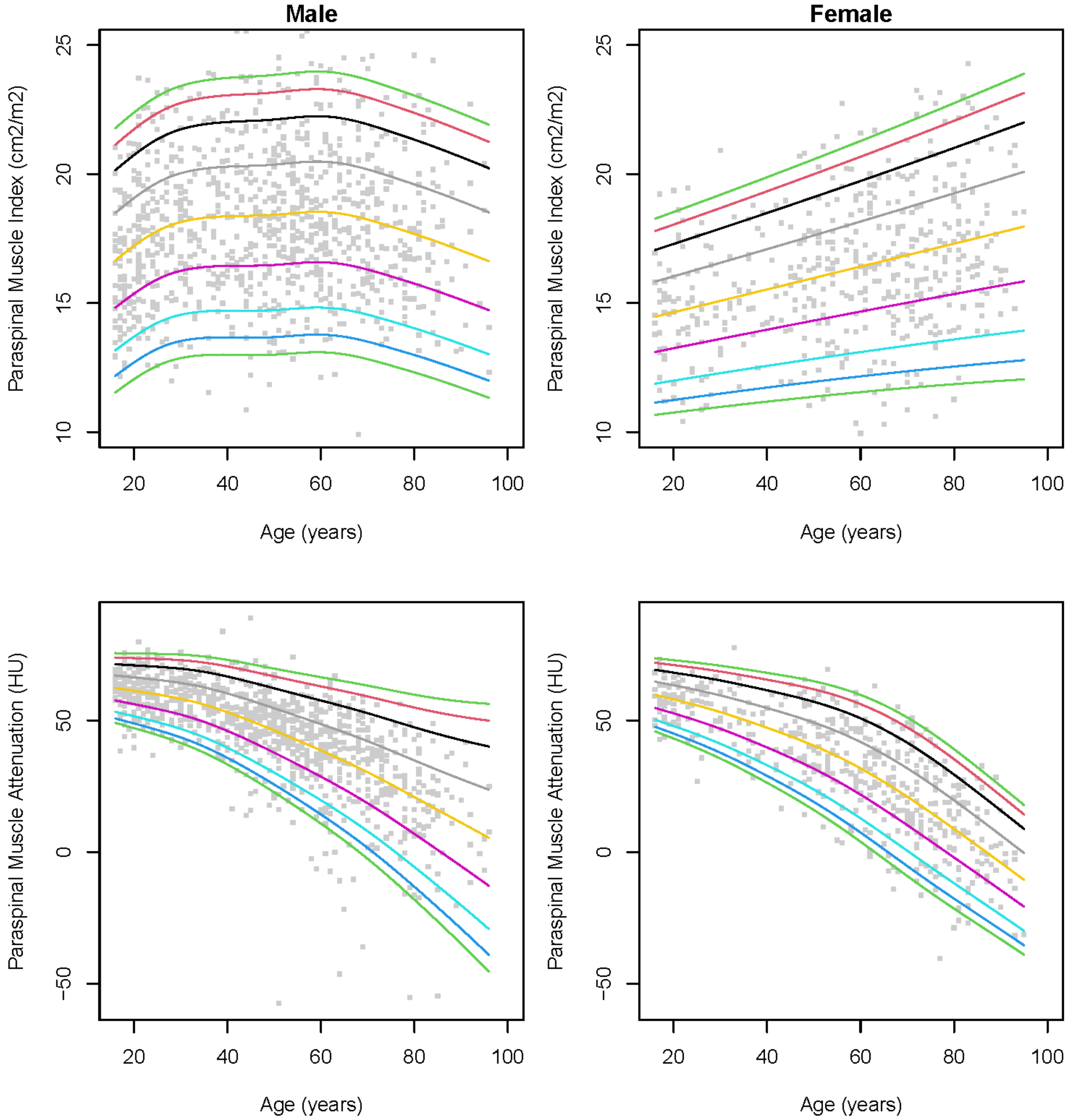

Supplemental figure 7. Reference curves of paraspinal muscle index and paraspinal muscle attenuation (Hounsfield units) of the complete areas over age in males and females. The colored lines represent the 3rd (dark green), 5th (dark blue), 10th (light blue), 25th (purple), 50th (yellow), 75th (grey), 90th (black), 95th (red), and 97th (light green) percentiles.

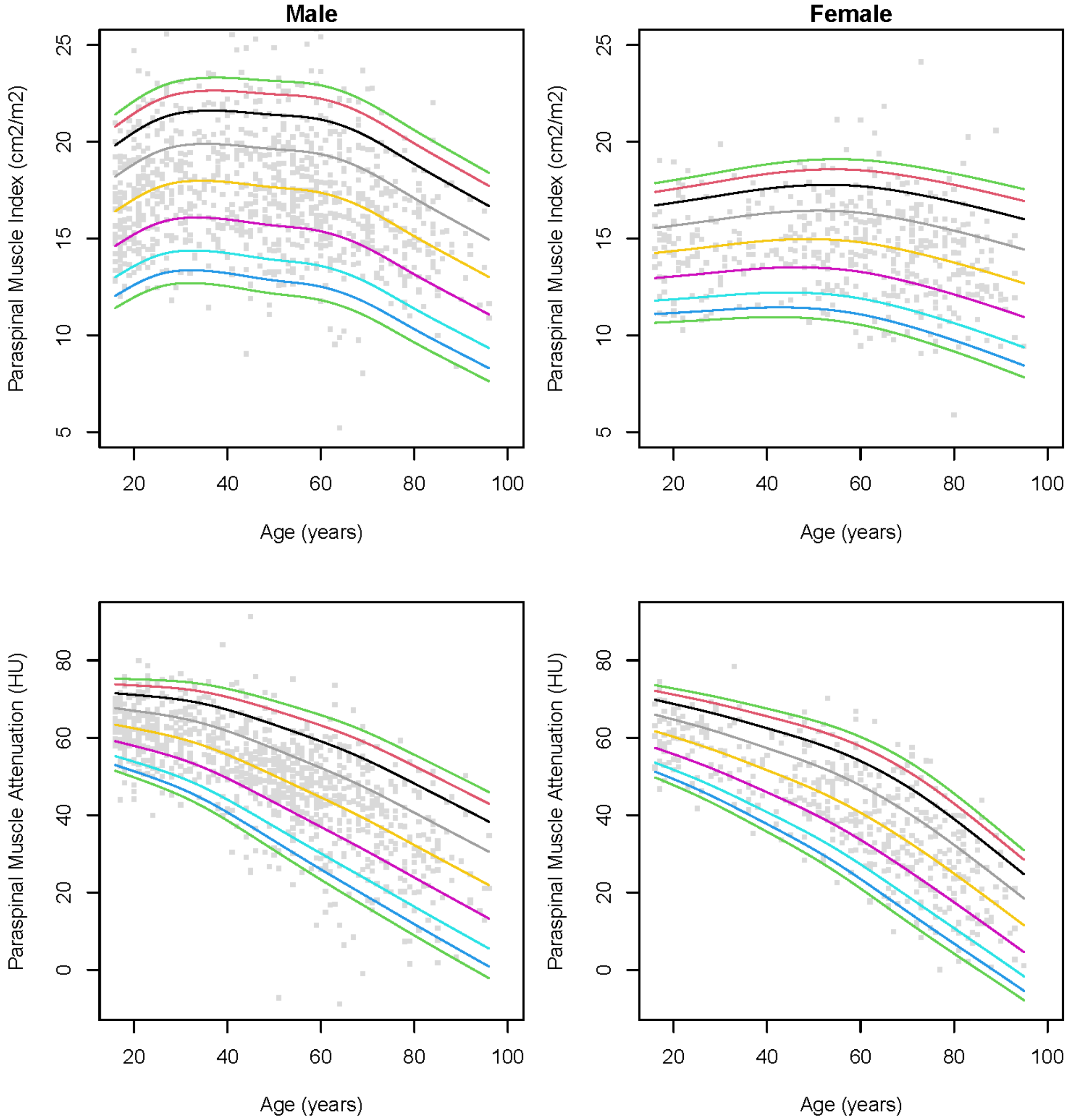

Supplemental figure 8. Reference curves of paraspinal muscle index and paraspinal muscle attenuation (Hounsfield units) of the pure-muscle sub-areas over age in males and females. The colored lines represent the 3rd (dark green), 5th (dark blue), 10th (light blue), 25th (purple), 50th (yellow), 75th (grey), 90th (black), 95th (red), and 97th (light green) percentiles.

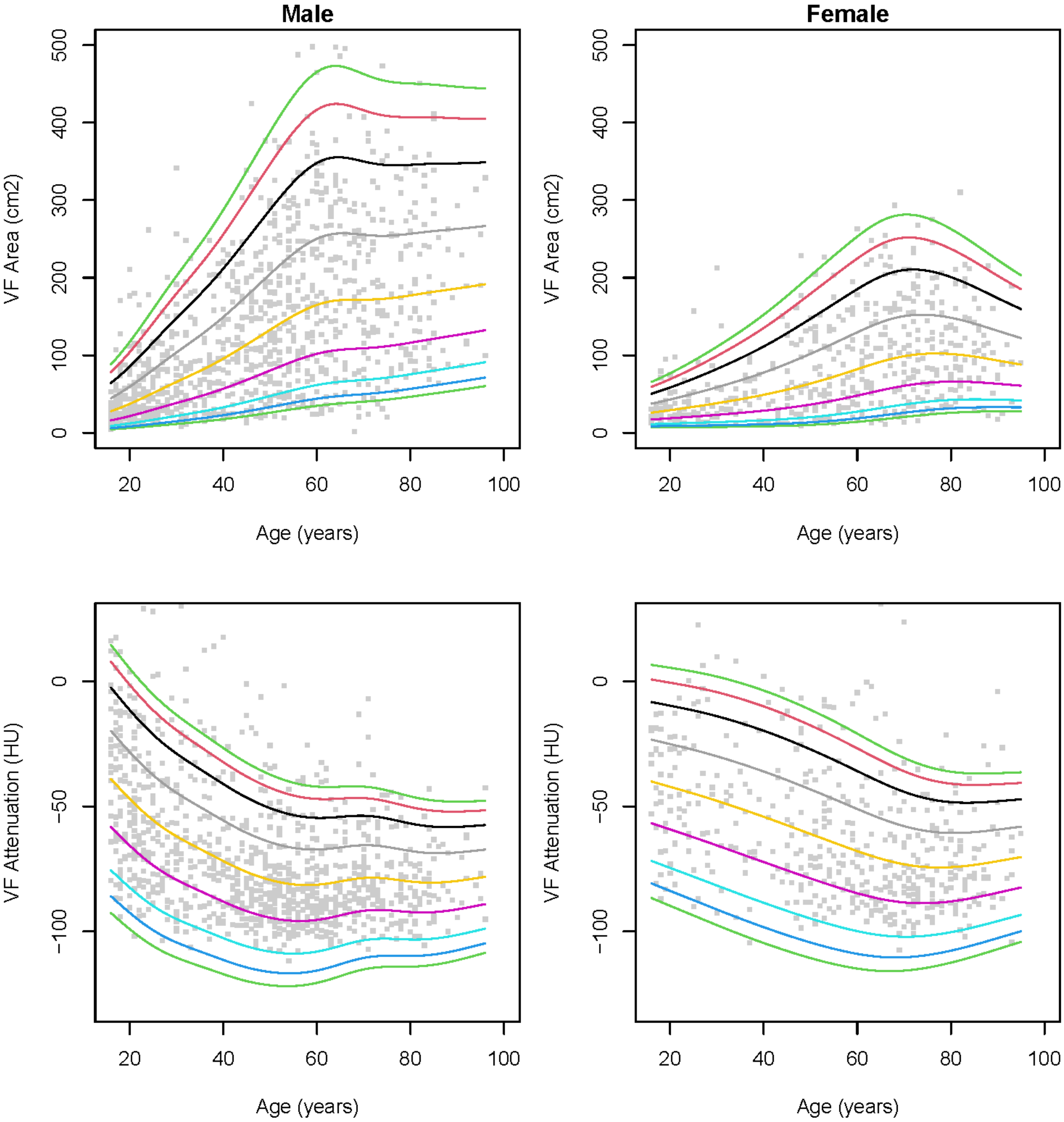

Supplemental figure 9. Reference curves of visceral fat (VF) area and visceral fat attenuation (Hounsfield units) over age in males and females. The colored lines represent the 3rd (dark green), 5th (dark blue), 10th (light blue), 25th (purple), 50th (yellow), 75th (grey), 90th (black), 95th (red), and 97th (light green) percentiles.

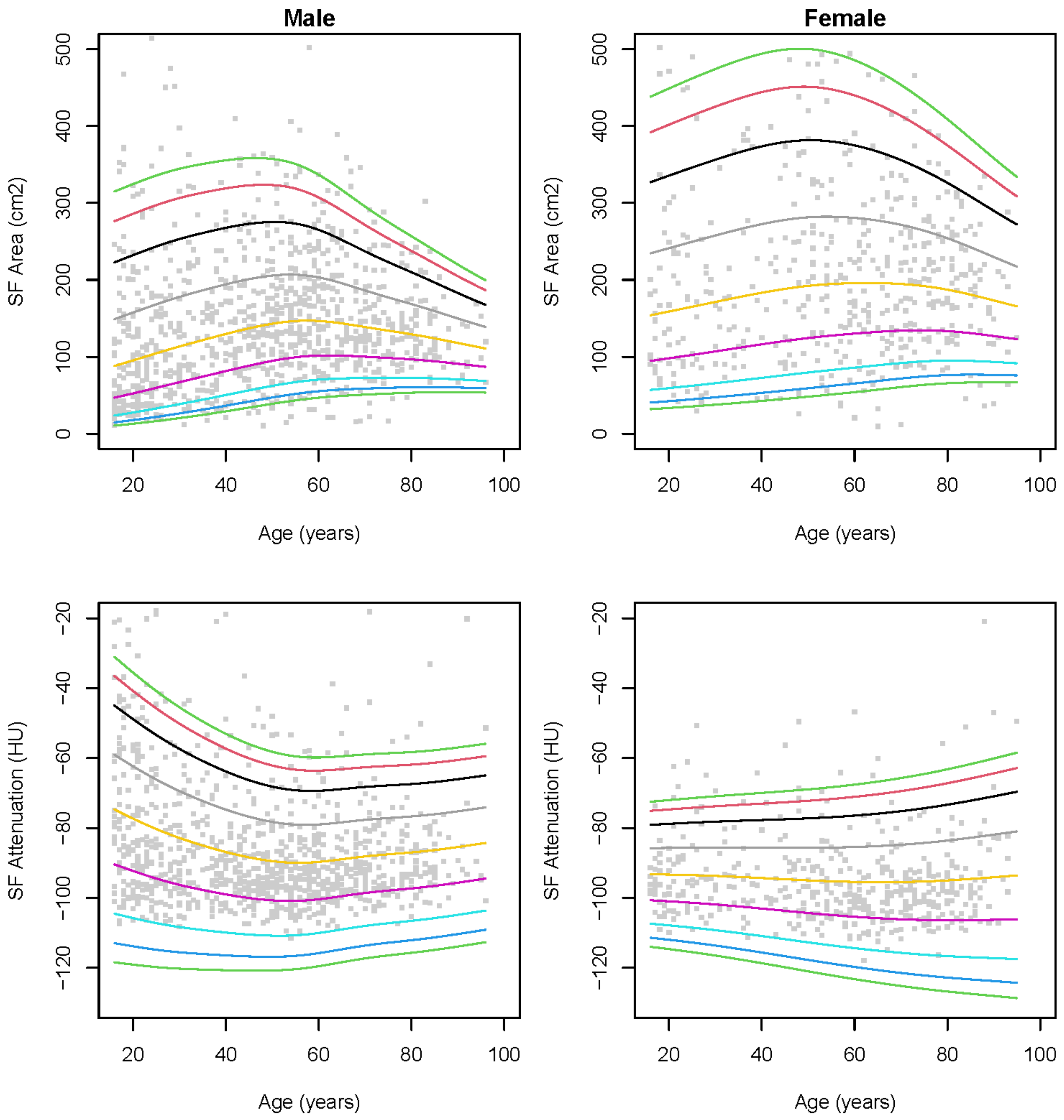

Supplemental figure 10. Reference curves of subcutaneous fat (SF) area and subcutaneous fat attenuation (Hounsfield units) over age in males and females. The colored lines represent the 3rd (dark green), 5th (dark blue), 10th (light blue), 25th (purple), 50th (yellow), 75th (grey), 90th (black), 95th (red), and 97th (light green) percentiles.

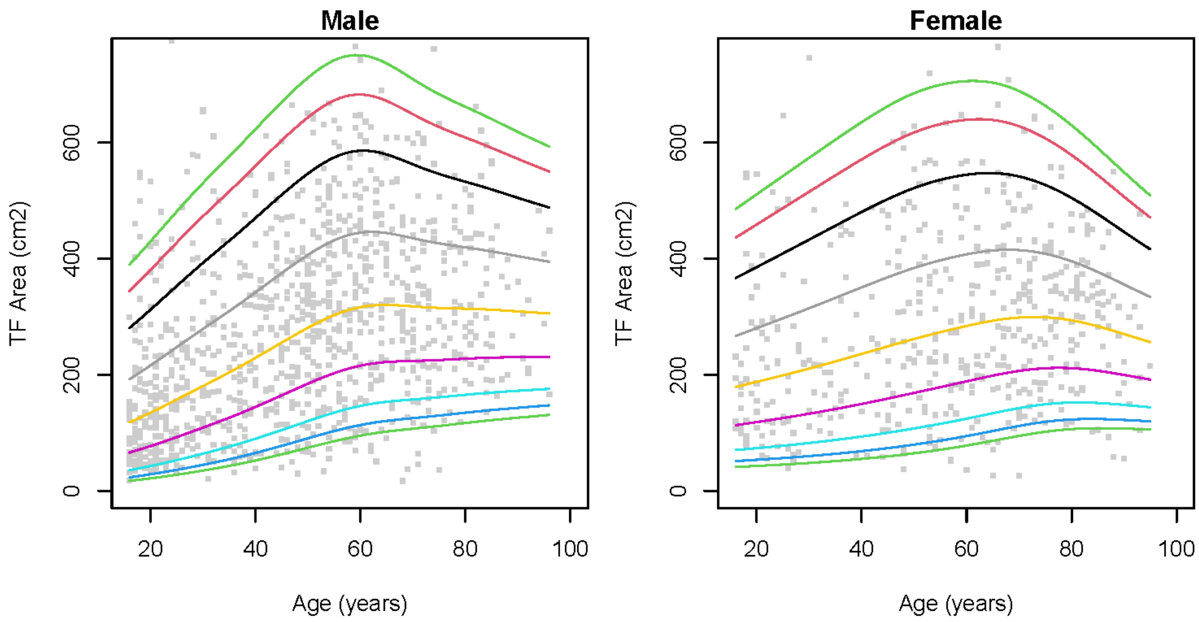

Supplemental figure 11. Reference curves of total fat (TF) area over age in males and females. The colored lines represent the 3rd (dark green), 5th (dark blue), 10th (light blue), 25th (purple), 50th (yellow), 75th (grey), 90th (black), 95th (red), and 97th (light green) percentiles.

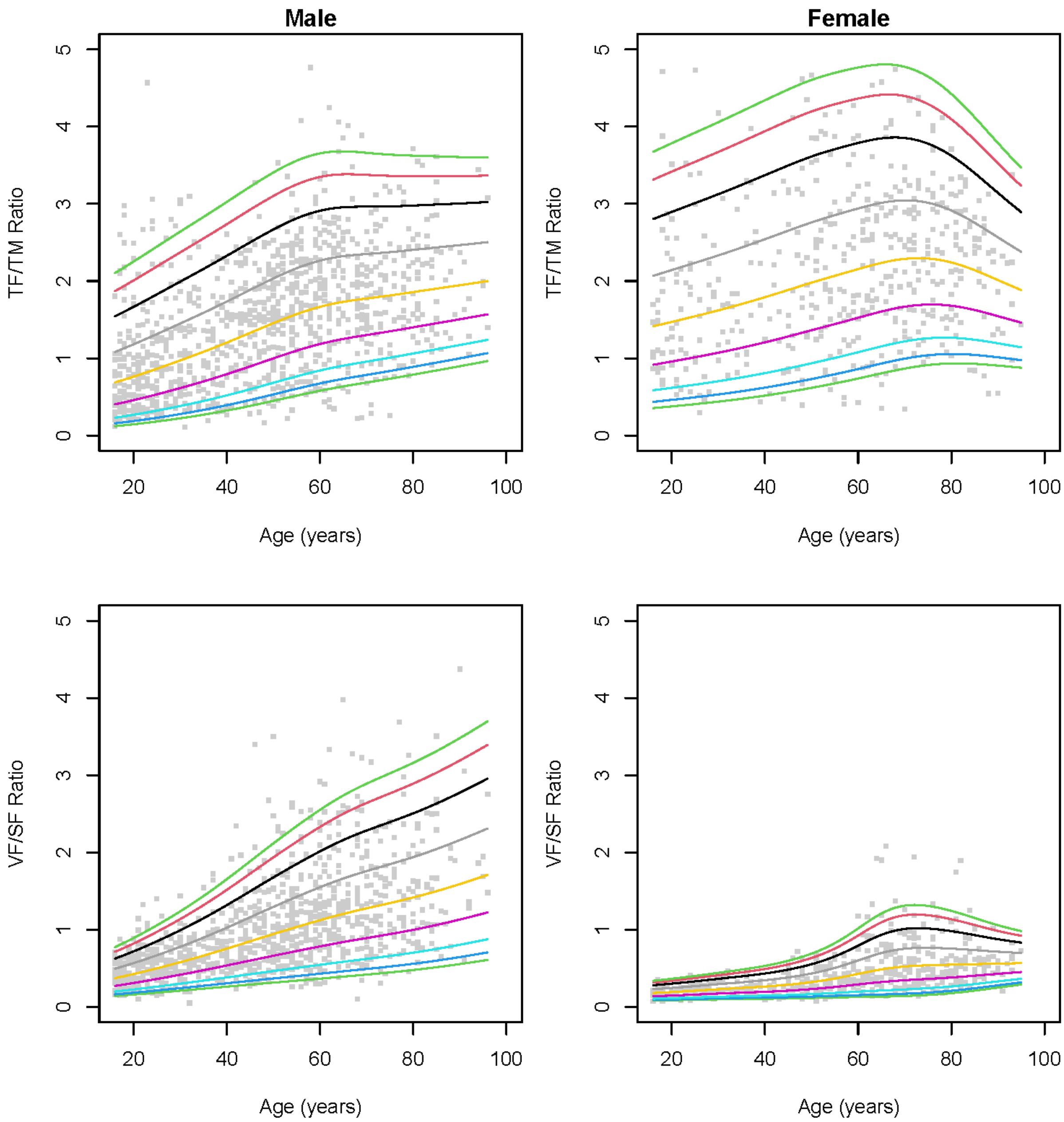

Supplemental figure 12. Reference curves of total fat to total muscle (TF/TM) ratio and visceral to subcutaneous fat (VF/SF) ratio over age in males and females. The colored lines represent the 3rd (dark green), 5th (dark blue), 10th (light blue), 25th (purple), 50th (yellow), 75th (grey), 90th (black), 95th (red), and 97th (light green) percentiles.

Supplemental tables

Supplemental Table 1. Percentiles of total muscle index (cm2/m2) based on complete muscle areas in males									
Age (years)	p3	p5	p10	p25	p50	p75	p90	p95	p97
20	38,3	40,1	42,9	47,5	52,7	57,9	62,5	65,3	67,1
25	40,3	42,2	45,1	50	55,4	60,8	65,7	68,6	70,5
30	41	42,9	46	51	56,7	62,3	67,4	70,4	72,4
35	40,9	42,9	46	51,3	57	62,8	68	71,2	73,2
40	40,8	42,9	46,1	51,4	57,3	63,2	68,6	71,7	73,8
45	40,8	42,9	46,1	51,6	57,6	63,7	69,1	72,4	74,5
50	40,7	42,9	46,2	51,7	57,9	64,1	69,7	73	75,2
55	40,5	42,7	46,1	51,8	58,1	64,4	70	73,4	75,6
60	40,1	42,3	45,7	51,4	57,8	64,1	69,8	73,3	75,5
65	39,7	41,9	45,3	50,9	57,1	63,4	69	72,4	74,6
70	39,6	41,7	44,9	50,4	56,4	62,5	67,9	71,2	73,3
75	39,7	41,7	44,8	50,1	55,8	61,6	66,8	69,9	72
80	40,1	42	45	49,9	55,5	61	66	68,9	70,9
85	40,4	42,3	45,1	49,9	55,1	60,4	65,2	68	69,9
90	40,5	42,3	45	49,6	54,7	59,8	64,3	67,1	68,8

Supplemental Table 2. Percentiles of total muscle index (cm2/m2) based on pure-muscle sub-areas in males									
Age (years)	p3	p5	p10	p25	p50	p75	p90	p95	p97
20	37,5	39,2	41,9	46,3	51,1	56	60,4	63	64,7
25	39,3	41	43,8	48,4	53,5	58,5	63,1	65,9	67,6
30	39,6	41,4	44,2	49	54,3	59,5	64,3	67,1	69
35	39,1	40,9	43,9	48,7	54,1	59,6	64,4	67,3	69,2
40	38,5	40,4	43,4	48,4	53,9	59,4	64,4	67,4	69,3
45	38	40	43	48	53,7	59,3	64,3	67,4	69,3
50	37,6	39,6	42,6	47,8	53,5	59,2	64,3	67,4	69,4
55	37	39	42,1	47,3	53,1	58,8	64	67,1	69,1
60	36,2	38,2	41,3	46,5	52,2	57,9	63,1	66,2	68,2
65	35,3	37,3	40,3	45,3	51	56,6	61,6	64,6	66,6
70	34,6	36,4	39,3	44,2	49,6	55	59,8	62,7	64,6
75	33,7	35,5	38,3	43	48,1	53,3	57,9	60,7	62,5
80	33	34,7	37,4	41,8	46,7	51,6	56,1	58,7	60,4
85	32,4	34	36,6	40,8	45,5	50,2	54,5	57	58,7
90	31,8	33,4	35,8	39,9	44,4	48,9	53	55,4	57

Supplemental Table 3. Percentiles of total muscle index (cm2/m2) based on complete muscle areas in females									
Age (years)	p3	p5	p10	p25	p50	p75	p90	p95	p97
20	33,1	34,6	36,9	40,7	44,9	49,1	52,9	55,2	56,6
25	33,2	34,7	37	40,9	45,1	49,4	53,3	55,6	57,1
30	33,3	34,8	37,1	41	45,4	49,7	53,7	56	57,5
35	33,3	34,8	37,2	41,2	45,6	50	54	56,4	58
40	33,3	34,9	37,3	41,4	45,8	50,3	54,4	56,8	58,4
45	33,3	34,9	37,4	41,5	46,1	50,6	54,7	57,2	58,8
50	33,3	35	37,5	41,7	46,3	50,9	55,1	57,6	59,2
55	33,4	35	37,6	41,8	46,5	51,3	55,5	58,1	59,7
60	33,5	35,1	37,7	42	46,8	51,6	55,9	58,5	60,2
65	33,6	35,3	37,9	42,3	47,2	52	56,4	59,1	60,8
70	33,7	35,5	38,1	42,6	47,5	52,5	57	59,6	61,4
75	33,9	35,7	38,4	42,9	48	53	57,5	60,2	62
80	34,2	35,9	38,7	43,3	48,4	53,5	58,1	60,9	62,7
85	34,4	36,2	39	43,7	48,9	54,1	58,8	61,6	63,4
90	34,6	36,5	39,3	44,1	49,4	54,7	59,4	62,3	64,1

Supplemental Table 4. Percentiles of total muscle index (cm2/m2) based on pure-muscle sub-areas in females									
Age (years)	p3	p5	p10	p25	p50	p75	p90	p95	p97
20	32,6	34	36,1	39,6	43,5	47,4	50,9	53,1	54,4
25	32,4	33,8	35,9	39,4	43,3	47,2	50,7	52,8	54,2
30	32,2	33,6	35,7	39,2	43,1	47	50,5	52,6	53,9
35	32	33,4	35,5	38,9	42,8	46,7	50,2	52,3	53,6
40	31,7	33,1	35,2	38,6	42,5	46,4	49,8	51,9	53,3
45	31,4	32,8	34,8	38,3	42,1	46	49,5	51,5	52,9
50	31	32,4	34,4	37,9	41,7	45,6	49	51,1	52,4
55	30,6	31,9	34	37,4	41,3	45,1	48,5	50,6	51,9
60	30,2	31,5	33,5	37	40,8	44,6	48	50,1	51,4
65	29,7	31	33,1	36,5	40,3	44,1	47,5	49,5	50,9
70	29,2	30,5	32,6	36	39,8	43,5	47	49	50,3
75	28,7	30,1	32,1	35,5	39,3	43	46,4	48,5	49,8
80	28,3	29,6	31,6	35	38,8	42,5	45,9	47,9	49,2
85	27,8	29,1	31,2	34,5	38,3	42	45,4	47,4	48,7
90	27,4	28,7	30,7	34,1	37,8	41,5	44,9	46,9	48,2

**Supplemental Table 5. Percentiles of total muscle attenuation (Hounsfield units) based on complete muscle areas in males**									
Age (years)	p3	p5	p10	p25	p50	p75	p90	p95	p97
20	34,3	36,2	39	43,7	48,9	54,2	58,9	61,7	63,6
25	31,2	33,2	36,1	41,1	46,6	52,1	57,1	60	62
30	28,1	30,1	33,2	38,4	44,2	50	55,2	58,3	60,4
35	24,8	26,9	30,2	35,7	41,8	47,8	53,3	56,6	58,7
40	21,4	23,6	27,1	32,8	39,2	45,6	51,4	54,8	57,1
45	17,9	20,2	23,9	29,9	36,6	43,4	49,4	53	55,4
50	14,3	16,8	20,6	26,9	34	41,1	47,4	51,2	53,7
55	10,6	13,2	17,2	23,8	31,3	38,7	45,4	49,4	52
60	6,7	9,5	13,7	20,7	28,5	36,3	43,3	47,5	50,3
65	2,8	5,6	10	17,4	25,6	33,8	41,2	45,6	48,5
70	-1,4	1,7	6,3	14,1	22,7	31,3	39,1	43,7	46,7
75	-5,6	-2,4	2,5	10,6	19,7	28,7	36,9	41,8	45
80	-9,9	-6,6	-1,5	7,1	16,6	26,1	34,7	39,8	43,2
85	-14,4	-10,9	-5,5	3,5	13,5	23,5	32,6	37,9	41,4
90	-18,9	-15,2	-9,6	-0,1	10,4	21	30,4	36,1	39,8

**Supplemental Table 6. Percentiles of total muscle attenuation (Hounsfield units) based on pure-muscle sub-areas in males**									
Age (years)	p3	p5	p10	p25	p50	p75	p90	p95	p97
20	41	42,3	44,4	48	51,9	55,8	59,3	61,4	62,8
25	38,9	40,3	42,5	46,1	50,2	54,2	57,9	60,1	61,5
30	36,8	38,2	40,5	44,3	48,5	52,7	56,4	58,7	60,2
35	34,6	36,1	38,4	42,3	46,7	51,1	55	57,3	58,8
40	32,3	33,9	36,3	40,4	44,9	49,4	53,5	55,9	57,5
45	30	31,6	34,1	38,3	43	47,7	51,9	54,4	56,1
50	27,6	29,3	31,9	36,3	41,1	46	50,3	52,9	54,6
55	25,1	26,9	29,6	34,1	39,2	44,2	48,7	51,4	53,2
60	22,6	24,5	27,3	32	37,2	42,4	47,1	49,9	51,7
65	20,1	22	24,9	29,7	35,1	40,5	45,4	48,3	50,2
70	17,4	19,4	22,4	27,4	33	38,6	43,7	46,7	48,6
75	14,7	16,7	19,9	25,1	30,9	36,7	41,9	45	47,1
80	11,9	14	17,3	22,7	28,7	34,7	40,1	43,4	45,5
85	9,1	11,3	14,7	20,3	26,5	32,7	38,3	41,7	43,9
90	6,3	8,5	12	17,8	24,3	30,8	36,6	40	42,3

Supplemental Table 7. Percentiles of total muscle attenuation (Hounsfield units) based on complete muscle areas in females									
Age (years)	p3	p5	p10	p25	p50	p75	p90	p95	p97
20	32,4	34,2	37,1	41,9	47,3	52,6	57,4	60,3	62,1
25	28,7	30,8	33,8	39	44,8	50,5	55,7	58,8	60,8
30	24,9	27,1	30,4	36	42,2	48,3	53,9	57,2	59,4
35	21	23,3	26,9	32,8	39,5	46,1	52,1	55,7	58
40	16,9	19,4	23,2	29,6	36,7	43,8	50,2	54,1	56,5
45	12,7	15,4	19,5	26,3	33,8	41,4	48,2	52,3	54,9
50	8,6	11,4	15,7	22,8	30,8	38,7	45,9	50,2	53
55	4,5	7,4	11,8	19,3	27,5	35,8	43,2	47,7	50,6
60	0,3	3,3	7,9	15,5	24	32,5	40,2	44,8	47,7
65	-3,9	-0,9	3,8	11,6	20,3	29	36,8	41,4	44,5
70	-8	-5	-0,3	7,6	16,4	25,1	33	37,7	40,8
75	-12	-8,9	-4,2	3,6	12,3	21,1	28,9	33,6	36,7
80	-15,8	-12,8	-8,1	-0,4	8,2	16,8	24,6	29,2	32,3
85	-19,5	-16,5	-12	-4,4	4,1	12,5	20,1	24,7	27,6
90	-23,1	-20,2	-15,7	-8,3	-0,1	8,2	15,6	20,1	22,9

Supplemental Table 8. Percentiles of total muscle attenuation (Hounsfield units) based on pure-muscle sub-areas in females									
Age (years)	p3	p5	p10	p25	p50	p75	p90	p95	p97
20	40	41,4	43,5	47,1	51,1	55	58,6	60,7	62,1
25	37,6	39,1	41,3	45	49,2	53,4	57,1	59,3	60,8
30	35,1	36,6	39	42,9	47,3	51,7	55,6	57,9	59,5
35	32,5	34,1	36,6	40,7	45,3	49,9	54	56,5	58,1
40	29,8	31,5	34,1	38,5	43,3	48,1	52,5	55,1	56,7
45	27,2	28,9	31,6	36,2	41,2	46,3	50,8	53,5	55,3
50	24,5	26,3	29,1	33,8	39,1	44,3	49	51,8	53,7
55	21,8	23,6	26,5	31,4	36,8	42,2	47,1	50	51,9
60	19	20,9	23,9	28,9	34,4	40	44,9	47,9	49,9
65	16,1	18,1	21,2	26,2	31,9	37,5	42,6	45,7	47,7
70	13,4	15,4	18,5	23,6	29,3	35	40,1	43,2	45,2
75	10,8	12,8	15,8	20,9	26,6	32,2	37,3	40,4	42,4
80	8,3	10,3	13,3	18,3	23,8	29,4	34,3	37,3	39,3
85	6	7,9	10,8	15,6	21	26,4	31,2	34,1	36
90	3,6	5,4	8,2	12,9	18,1	23,3	28	30,8	32,7

Supplemental Table 9. Percentiles of psoas muscle index (cm2/m2) based on complete muscle areas in males									
Age (years)	p3	p5	p10	p25	p50	p75	p90	p95	p97
20	4,5	4,9	5,4	6,4	7,4	8,4	9,4	10	10,3
25	4,7	5,1	5,6	6,6	7,7	8,7	9,7	10,2	10,6
30	4,7	5,1	5,6	6,6	7,7	8,7	9,7	10,3	10,7
35	4,5	4,9	5,5	6,4	7,5	8,6	9,6	10,1	10,5
40	4,4	4,7	5,3	6,3	7,4	8,4	9,4	10	10,4
45	4,3	4,7	5,2	6,2	7,3	8,4	9,3	9,9	10,3
50	4,2	4,6	5,2	6,2	7,2	8,3	9,3	9,8	10,2
55	4,2	4,5	5,1	6,1	7,1	8,2	9,1	9,7	10,1
60	4	4,3	4,9	5,8	6,9	7,9	8,9	9,4	9,8
65	3,8	4,1	4,7	5,6	6,6	7,6	8,5	9,1	9,4
70	3,6	3,9	4,5	5,3	6,3	7,3	8,2	8,7	9
75	3,5	3,8	4,3	5,2	6,1	7	7,9	8,4	8,7
80	3,5	3,8	4,3	5,1	5,9	6,8	7,6	8,1	8,4
85	3,5	3,8	4,2	5	5,8	6,7	7,4	7,9	8,2
90	3,5	3,7	4,2	4,9	5,7	6,5	7,2	7,6	7,9

Supplemental Table 10. Percentiles of psoas muscle index (cm2/m2) based on pure-muscle sub-areas in males									
Age (years)	p3	p5	p10	p25	p50	p75	p90	p95	p97
20	4,3	4,7	5,3	6,2	7,3	8,4	9,4	9,9	10,3
25	4,5	4,8	5,4	6,4	7,5	8,6	9,6	10,2	10,5
30	4,4	4,8	5,4	6,4	7,5	8,6	9,6	10,2	10,6
35	4,2	4,6	5,2	6,2	7,3	8,4	9,4	10	10,4
40	4	4,4	5	6	7,2	8,3	9,3	9,9	10,3
45	3,9	4,3	4,9	5,9	7,1	8,2	9,2	9,8	10,2
50	3,9	4,2	4,8	5,9	7	8,1	9,1	9,7	10,1
55	3,8	4,1	4,7	5,7	6,8	8	9	9,6	9,9
60	3,6	3,9	4,5	5,5	6,6	7,7	8,7	9,3	9,7
65	3,4	3,7	4,3	5,3	6,3	7,4	8,3	8,9	9,3
70	3,2	3,6	4,1	5	6,1	7,1	8	8,5	8,9
75	3,1	3,5	4	4,8	5,8	6,8	7,6	8,2	8,5
80	3	3,4	3,9	4,7	5,6	6,5	7,3	7,8	8,1
85	3	3,3	3,8	4,6	5,4	6,3	7,1	7,5	7,9
90	3	3,3	3,7	4,5	5,3	6,1	6,8	7,3	7,6

Supplemental Table 11. Percentiles of psoas muscle index (cm2/m2) based on complete muscle areas in females									
Age (years)	p3	p5	p10	p25	p50	p75	p90	p95	p97
20	3,6	3,9	4,3	5	5,7	6,5	7,2	7,6	7,8
25	3,5	3,8	4,2	4,9	5,6	6,4	7	7,5	7,7
30	3,4	3,7	4,1	4,8	5,5	6,3	6,9	7,3	7,6
35	3,3	3,6	4	4,7	5,4	6,1	6,8	7,2	7,5
40	3,2	3,5	3,9	4,5	5,3	6	6,7	7,1	7,3
45	3,1	3,4	3,8	4,4	5,1	5,9	6,5	6,9	7,2
50	3	3,3	3,6	4,3	5	5,8	6,4	6,8	7,1
55	2,9	3,2	3,5	4,2	4,9	5,6	6,3	6,7	6,9
60	2,8	3,1	3,5	4,1	4,8	5,5	6,2	6,6	6,8
65	2,8	3	3,4	4	4,7	5,5	6,1	6,5	6,7
70	2,7	3	3,3	4	4,7	5,4	6	6,4	6,7
75	2,7	2,9	3,3	3,9	4,6	5,3	6	6,4	6,6
80	2,7	2,9	3,3	3,9	4,6	5,3	5,9	6,3	6,6
85	2,7	2,9	3,3	3,9	4,6	5,3	5,9	6,3	6,5
90	2,7	2,9	3,3	3,9	4,6	5,3	5,9	6,3	6,5

Supplemental Table 12. Percentiles of psoas muscle index (cm2/m2) based on pure-muscle sub-areas in females									
Age (years)	p3	p5	p10	p25	p50	p75	p90	p95	p97
20	3,5	3,8	4,2	4,9	5,7	6,4	7,1	7,5	7,8
25	3,4	3,7	4,1	4,8	5,5	6,3	7	7,4	7,7
30	3,3	3,6	4	4,7	5,4	6,2	6,9	7,3	7,5
35	3,2	3,5	3,9	4,5	5,3	6,1	6,7	7,1	7,4
40	3,1	3,3	3,7	4,4	5,2	5,9	6,6	7	7,3
45	3	3,2	3,6	4,3	5,1	5,8	6,5	6,9	7,1
50	2,8	3,1	3,5	4,2	4,9	5,7	6,3	6,7	7
55	2,7	3	3,4	4,1	4,8	5,6	6,2	6,6	6,9
60	2,6	2,9	3,3	4	4,7	5,4	6,1	6,5	6,8
65	2,6	2,8	3,2	3,9	4,6	5,3	6	6,4	6,6
70	2,5	2,7	3,1	3,8	4,5	5,2	5,9	6,3	6,5
75	2,4	2,6	3	3,7	4,4	5,1	5,8	6,2	6,4
80	2,3	2,6	3	3,6	4,3	5,1	5,7	6,1	6,3
85	2,2	2,5	2,9	3,5	4,3	5	5,6	6	6,3
90	2,2	2,4	2,8	3,5	4,2	4,9	5,5	5,9	6,2

Supplemental Table 13. Percentiles of psoas muscle attenuation (Hounsfield units) based on complete muscle areas in males									
Age (years)	p3	p5	p10	p25	p50	p75	p90	p95	p97
20	19,9	23,3	28,5	37,1	46,7	56,3	65	70,2	73,5
25	18,2	21,6	26,8	35,6	45,3	55	63,7	68,9	72,3
30	16,5	19,9	25,2	34	43,8	53,6	62,4	67,7	71,1
35	14,7	18,2	23,5	32,4	42,3	52,2	61,1	66,4	69,9
40	13	16,5	21,9	30,8	40,8	50,8	59,8	65,2	68,7
45	11,2	14,8	20,2	29,3	39,4	49,4	58,5	64	67,5
50	9,5	13,1	18,5	27,7	37,9	48,1	57,2	62,7	66,3
55	7,7	11,3	16,9	26,1	36,4	46,7	55,9	61,5	65,1
60	6	9,6	15,2	24,6	34,9	45,3	54,7	60,2	63,9
65	4,2	7,9	13,6	23	33,5	43,9	53,4	59	62,7
70	2,5	6,2	11,9	21,4	32	42,6	52,1	57,8	61,5
75	0,7	4,5	10,2	19,8	30,5	41,2	50,8	56,6	60,3
80	-1	2,8	8,6	18,3	29	39,8	49,5	55,3	59,1
85	-2,8	1	6,9	16,7	27,6	38,4	48,2	54,1	57,9
90	-4,5	-0,7	5,2	15,1	26,1	37,1	47	52,9	56,7

Supplemental Table 14. Percentiles of psoas muscle attenuation (Hounsfield units) based on pure-muscle sub-areas in males									
Age (years)	p3	p5	p10	p25	p50	p75	p90	p95	p97
20	24,4	27,3	31,9	39,4	47,8	56,2	63,8	68,3	71,3
25	23,3	26,2	30,7	38,2	46,6	55	62,5	67	70
30	22,1	25	29,5	37	45,4	53,7	61,2	65,7	68,7
35	21	23,9	28,4	35,9	44,2	52,5	60	64,4	67,4
40	19,9	22,7	27,2	34,7	43	51,2	58,7	63,2	66
45	18,7	21,6	26	33,5	41,7	50	57,4	61,9	64,7
50	17,6	20,5	24,9	32,3	40,5	48,7	56,1	60,6	63,4
55	16,4	19,3	23,7	31,1	39,3	47,5	54,9	59,3	62,1
60	15,3	18,2	22,6	29,9	38,1	46,2	53,6	58	60,8
65	14,2	17	21,4	28,7	36,9	45	52,3	56,7	59,5
70	13	15,9	20,2	27,5	35,6	43,7	51	55,4	58,2
75	11,9	14,7	19,1	26,3	34,4	42,5	49,8	54,1	56,9
80	10,8	13,6	17,9	25,2	33,2	41,2	48,5	52,8	55,6
85	9,6	12,4	16,7	24	32	40	47,2	51,5	54,3
90	8,5	11,3	15,6	22,8	30,8	38,8	45,9	50,2	53

Supplemental Table 15. Percentiles of psoas muscle attenuation (Hounsfield units) based on complete muscle areas in females									
Age (years)	p3	p5	p10	p25	p50	p75	p90	p95	p97
20	32,7	35,2	39,1	45,6	52,8	60	66,5	70,4	73
25	30,2	32,8	36,8	43,5	51	58,4	65,1	69,1	71,7
30	27,8	30,4	34,6	41,4	49,1	56,7	63,6	67,8	70,4
35	25,2	28	32,2	39,3	47,2	55,1	62,2	66,4	69,2
40	22,7	25,6	29,9	37,2	45,4	53,5	60,8	65,1	68
45	20,2	23,1	27,6	35,1	43,5	51,8	59,4	63,9	66,8
50	17,6	20,6	25,3	33	41,6	50,2	58	62,6	65,6
55	15	18,1	22,9	30,9	39,7	48,6	56,6	61,4	64,5
60	12,4	15,6	20,5	28,8	37,9	47	55,2	60,1	63,3
65	9,8	13,1	18,2	26,6	36	45,4	53,9	58,9	62,2
70	7,2	10,5	15,8	24,5	34,1	43,8	52,5	57,7	61,1
75	4,5	8	13,3	22,3	32,3	42,2	51,2	56,6	60
80	1,8	5,4	10,9	20,1	30,4	40,7	49,9	55,4	59
85	-0,9	2,8	8,5	18	28,5	39,1	48,6	54,3	58
90	-3,7	0,1	6	15,8	26,7	37,5	47,3	53,2	57

Supplemental Table 16. Percentiles of psoas muscle attenuation (Hounsfield units) based on pure-muscle sub-areas in females									
Age (years)	p3	p5	p10	p25	p50	p75	p90	p95	p97
20	34,6	36,9	40,5	46,5	53,2	59,9	65,9	69,5	71,8
25	32,8	35,1	38,8	44,9	51,7	58,4	64,5	68,2	70,5
30	31	33,4	37,1	43,3	50,1	57	63,1	66,8	69,2
35	29,2	31,6	35,4	41,6	48,6	55,5	61,8	65,5	67,9
40	27,4	29,9	33,7	40	47	54,1	60,4	64,2	66,6
45	25,6	28,1	32	38,4	45,5	52,6	59	62,9	65,3
50	23,8	26,4	30,2	36,7	43,9	51,2	57,6	61,5	64,1
55	22	24,6	28,5	35,1	42,4	49,7	56,3	60,2	62,8
60	20,2	22,8	26,8	33,5	40,9	48,3	54,9	58,9	61,5
65	18,4	21,1	25,1	31,8	39,3	46,8	53,6	57,6	60,2
70	16,6	19,3	23,4	30,2	37,8	45,4	52,2	56,3	58,9
75	14,8	17,5	21,6	28,6	36,2	43,9	50,8	55	57,7
80	13	15,7	19,9	26,9	34,7	42,5	49,5	53,7	56,4
85	11,2	13,9	18,2	25,3	33,2	41	48,1	52,4	55,1
90	9,4	12,2	16,5	23,6	31,6	39,6	46,8	51,1	53,9

Supplemental Table 17. Percentiles of abdominal muscle index (cm2/m2) based on complete muscle areas in males									
Age (years)	p3	p5	p10	p25	p50	p75	p90	p95	p97
20	18,9	20,1	21,9	24,8	28,1	31,5	34,4	36,2	37,4
25	20	21,2	23,1	26,2	29,7	33,2	36,4	38,3	39,5
30	20,5	21,7	23,7	27	30,7	34,3	37,6	39,6	40,9
35	20,5	21,9	23,9	27,4	31,2	35	38,4	40,5	41,8
40	20,6	22	24,1	27,7	31,6	35,6	39,2	41,3	42,7
45	20,6	22	24,2	27,9	32	36,1	39,8	42	43,5
50	20,5	22	24,3	28,1	32,3	36,6	40,4	42,7	44,2
55	20,2	21,8	24,1	28,1	32,5	36,8	40,8	43,2	44,7
60	19,9	21,5	23,9	27,9	32,4	36,9	40,9	43,3	44,9
65	19,7	21,3	23,7	27,7	32,2	36,7	40,7	43,1	44,6
70	19,8	21,3	23,7	27,6	32	36,4	40,3	42,6	44,2
75	20,1	21,6	23,9	27,7	31,9	36,1	39,9	42,1	43,6
80	20,7	22,1	24,3	27,9	31,9	35,9	39,5	41,7	43,1
85	21,3	22,6	24,7	28,1	31,9	35,8	39,2	41,3	42,6
90	21,8	23,1	25	28,3	32	35,6	38,9	40,9	42,1

Supplemental Table 18. Percentiles of abdominal muscle index (cm2/m2) based on pure-muscle sub-areas in males									
Age (years)	p3	p5	p10	p25	p50	p75	p90	p95	p97
20	18,6	19,6	21,2	23,9	26,8	29,8	32,5	34,1	35,1
25	19,4	20,5	22,2	25	28,1	31,2	34,1	35,7	36,8
30	19,6	20,7	22,5	25,4	28,7	32	34,9	36,6	37,8
35	19,4	20,6	22,4	25,4	28,8	32,2	35,2	37,1	38,3
40	19,1	20,3	22,2	25,4	28,9	32,4	35,5	37,4	38,6
45	18,8	20,1	22	25,3	28,9	32,5	35,7	37,7	38,9
50	18,5	19,8	21,8	25,2	28,9	32,6	35,9	37,9	39,2
55	18,2	19,5	21,5	24,9	28,7	32,4	35,8	37,9	39,2
60	17,6	19	21	24,4	28,2	32	35,4	37,5	38,8
65	17,2	18,5	20,6	23,9	27,7	31,4	34,8	36,8	38,1
70	17	18,2	20,2	23,5	27,1	30,7	34	36	37,2
75	16,9	18,1	20	23,1	26,6	30	33,2	35	36,3
80	16,8	18	19,8	22,8	26,1	29,4	32,4	34,2	35,3
85	16,9	18	19,7	22,5	25,7	28,8	31,7	33,4	34,5
90	16,9	18	19,6	22,3	25,3	28,3	31	32,6	33,7

Supplemental Table 19. Percentiles of abdominal muscle index (cm2/m2) based on complete muscle areas in females									
Age (years)	p3	p5	p10	p25	p50	p75	p90	p95	p97
20	17,1	18	19,5	21,8	24,5	27,2	29,6	31	31,9
25	17	18	19,5	21,9	24,7	27,4	29,9	31,4	32,3
30	17	18	19,5	22	24,8	27,7	30,2	31,7	32,7
35	16,9	17,9	19,4	22,1	25	27,9	30,5	32	33,1
40	16,7	17,7	19,4	22,1	25,1	28,1	30,8	32,4	33,4
45	16,5	17,6	19,3	22,1	25,1	28,2	31	32,7	33,8
50	16,3	17,5	19,2	22	25,2	28,4	31,3	33	34,1
55	16,2	17,3	19,1	22	25,3	28,6	31,6	33,3	34,5
60	16	17,2	19	22,1	25,5	28,9	31,9	33,7	34,9
65	16	17,2	19,1	22,2	25,7	29,2	32,3	34,2	35,4
70	15,9	17,2	19,1	22,3	25,9	29,5	32,7	34,7	35,9
75	15,9	17,2	19,2	22,5	26,2	29,9	33,3	35,2	36,5
80	15,9	17,3	19,3	22,7	26,6	30,4	33,8	35,9	37,2
85	16	17,3	19,4	23	26,9	30,8	34,4	36,5	37,9
90	16	17,4	19,6	23,2	27,3	31,3	34,9	37,1	38,5

Supplemental Table 20. Percentiles of abdominal muscle index (cm2/m2) based on pure-muscle sub-areas in females									
Age (years)	p3	p5	p10	p25	p50	p75	p90	p95	p97
20	16,7	17,5	18,8	20,9	23,3	25,7	27,8	29,1	29,9
25	16,4	17,2	18,5	20,7	23,1	25,4	27,6	28,9	29,7
30	16,1	17	18,3	20,4	22,8	25,2	27,4	28,7	29,5
35	15,9	16,7	18	20,2	22,6	25	27,2	28,5	29,3
40	15,6	16,5	17,8	19,9	22,4	24,8	27	28,3	29,2
45	15,3	16,2	17,5	19,7	22,2	24,6	26,8	28,1	29
50	15,1	15,9	17,2	19,5	21,9	24,4	26,6	27,9	28,8
55	14,8	15,7	17	19,2	21,7	24,2	26,4	27,7	28,6
60	14,5	15,4	16,7	19	21,5	24	26,2	27,6	28,4
65	14,2	15,1	16,5	18,7	21,2	23,8	26	27,4	28,3
70	14	14,9	16,2	18,5	21	23,5	25,8	27,2	28,1
75	13,7	14,6	16	18,2	20,8	23,3	25,6	27	27,9
80	13,4	14,3	15,7	18	20,6	23,1	25,4	26,8	27,7
85	13,1	14,1	15,4	17,8	20,3	22,9	25,2	26,6	27,5
90	12,9	13,8	15,2	17,5	20,1	22,7	25	26,4	27,3

Supplemental Table 21. Percentiles of abdominal muscle attenuation (Hounsfield units) based on complete muscle areas in males									
Age (years)	p3	p5	p10	p25	p50	p75	p90	p95	p97
20	22,3	24,8	28,5	34,8	41,7	48,6	54,9	58,6	61,1
25	19,3	21,8	25,6	32	39,1	46,3	52,7	56,5	59
30	16,3	18,8	22,7	29,3	36,6	43,9	50,5	54,4	56,9
35	13,2	15,8	19,8	26,6	34,1	41,5	48,3	52,3	54,9
40	10,1	12,8	16,9	23,8	31,5	39,2	46,1	50,2	52,9
45	7	9,8	14	21,1	29	36,8	43,9	48,1	50,9
50	3,9	6,8	11,1	18,4	26,4	34,5	41,7	46,1	48,9
55	0,8	3,7	8,2	15,6	23,9	32,1	39,5	44	46,9
60	-2,3	0,7	5,2	12,8	21,3	29,8	37,4	42	44,9
65	-5,4	-2,4	2,3	10,1	18,8	27,4	35,2	39,9	43
70	-8,6	-5,5	-0,7	7,3	16,2	25,1	33,1	37,9	41
75	-11,7	-8,6	-3,6	4,6	13,7	22,8	31	35,9	39,1
80	-14,9	-11,7	-6,6	1,8	11,1	20,5	28,9	33,9	37,2
85	-18,1	-14,8	-9,6	-1	8,6	18,2	26,8	31,9	35,3
90	-21,3	-17,9	-12,6	-3,8	6	15,8	24,7	30	33,4

Supplemental Table 22. Percentiles of abdominal muscle attenuation (Hounsfield units) based on pure-muscle sub-areas in males									
Age (years)	p3	p5	p10	p25	p50	p75	p90	p95	p97
20	32,6	34,2	36,8	41,2	46	50,8	55,1	57,7	59,4
25	30,6	32,3	35	39,3	44,2	49,1	53,5	56,1	57,8
30	28,7	30,4	33,1	37,5	42,5	47,4	51,9	54,6	56,3
35	26,7	28,5	31,2	35,7	40,7	45,8	50,3	53	54,7
40	24,8	26,5	29,3	33,9	39	44,1	48,7	51,4	53,2
45	22,8	24,6	27,4	32	37,2	42,4	47	49,8	51,6
50	20,8	22,7	25,5	30,2	35,5	40,7	45,4	48,3	50,1
55	18,9	20,7	23,6	28,4	33,7	39	43,8	46,7	48,6
60	16,9	18,8	21,7	26,6	32	37,4	42,2	45,1	47
65	14,9	16,8	19,8	24,7	30,2	35,7	40,6	43,6	45,5
70	13	14,9	17,9	22,9	28,5	34	39	42	44
75	11	12,9	16	21,1	26,7	32,3	37,4	40,5	42,4
80	9	11	14,1	19,2	24,9	30,7	35,8	38,9	40,9
85	7	9	12,2	17,4	23,2	29	34,2	37,4	39,4
90	5	7,1	10,3	15,6	21,4	27,3	32,6	35,8	37,9

**Supplemental Table 23. Percentiles of abdominal muscle attenuation (Hounsfield units) based on complete muscle areas in females**									
Age (years)	p3	p5	p10	p25	p50	p75	p90	p95	p97
20	21,6	23,9	27,4	33,3	39,8	46,3	52,2	55,7	58
25	17,5	19,9	23,7	29,9	36,8	43,8	50	53,8	56,2
30	13,3	15,9	19,9	26,5	33,9	41,3	47,9	51,9	54,5
35	9,1	11,8	16,1	23,1	31	38,8	45,9	50,1	52,8
40	4,9	7,8	12,3	19,7	28	36,3	43,8	48,2	51,1
45	0,9	3,9	8,6	16,4	25,1	33,8	41,6	46,3	49,3
50	-3	0,2	5	13,1	22,1	31,1	39,2	44,1	47,2
55	-6,6	-3,3	1,6	10	19,2	28,4	36,7	41,7	44,9
60	-10	-6,7	-1,6	6,9	16,3	25,6	34,1	39,2	42,5
65	-13,2	-9,8	-4,7	3,8	13,3	22,8	31,3	36,5	39,8
70	-16,1	-12,8	-7,7	0,9	10,4	19,9	28,4	33,5	36,9
75	-18,9	-15,6	-10,5	-2	7,4	16,9	25,4	30,5	33,8
80	-21,6	-18,3	-13,3	-4,9	4,5	13,8	22,2	27,3	30,5
85	-24,2	-20,9	-16	-7,7	1,5	10,8	19,1	24	27,2
90	-26,7	-23,5	-18,6	-10,5	-1,4	7,7	15,8	20,7	23,9

**Supplemental Table 24. Percentiles of abdominal muscle attenuation (Hounsfield units) based on pure-muscle sub-areas in females**									
Age (years)	p3	p5	p10	p25	p50	p75	p90	p95	p97
20	32	33,6	36	40,1	44,7	49,2	53,4	55,8	57,4
25	29,4	31,1	33,6	37,9	42,7	47,4	51,7	54,3	55,9
30	26,8	28,6	31,2	35,7	40,6	45,6	50,1	52,7	54,5
35	24,2	26	28,8	33,5	38,6	43,8	48,4	51,2	53
40	21,7	23,5	26,4	31,2	36,6	42	46,8	49,7	51,6
45	19,1	21,1	24	29	34,6	40,1	45,1	48,1	50,1
50	16,7	18,7	21,7	26,9	32,6	38,3	43,4	46,5	48,5
55	14,3	16,3	19,5	24,7	30,5	36,4	41,6	44,7	46,8
60	12	14,1	17,3	22,6	28,5	34,4	39,8	43	45
65	9,8	11,9	15,1	20,5	26,5	32,5	37,9	41,1	43,2
70	7,8	9,9	13,1	18,5	24,5	30,5	35,9	39,1	41,2
75	5,8	7,9	11,1	16,5	22,5	28,4	33,8	37	39,1
80	4	6	9,2	14,5	20,5	26,4	31,7	34,9	36,9
85	2,2	4,2	7,4	12,6	18,4	24,3	29,5	32,6	34,7
90	0,4	2,4	5,5	10,7	16,4	22,1	27,3	30,4	32,4

Supplemental Table 25. Percentiles of paraspinal muscle index (cm2/m2) based on complete muscle areas in males									
Age (years)	p3	p5	p10	p25	p50	p75	p90	p95	p97
20	12	12,7	13,7	15,3	17,2	19	20,7	21,7	22,3
25	12,6	13,3	14,3	15,9	17,8	19,7	21,4	22,4	23
30	12,9	13,6	14,6	16,3	18,2	20,1	21,8	22,8	23,5
35	13,1	13,7	14,7	16,5	18,4	20,3	22	23	23,7
40	13,1	13,7	14,8	16,5	18,4	20,3	22	23,1	23,8
45	13	13,7	14,7	16,5	18,4	20,3	22,1	23,1	23,8
50	13	13,7	14,7	16,5	18,4	20,3	22,1	23,1	23,8
55	13	13,7	14,8	16,5	18,4	20,4	22,1	23,2	23,9
60	13,1	13,7	14,8	16,5	18,5	20,4	22,2	23,2	23,9
65	13	13,7	14,7	16,4	18,4	20,3	22,1	23,1	23,8
70	12,8	13,5	14,5	16,2	18,2	20,1	21,9	22,9	23,6
75	12,6	13,2	14,3	16	17,9	19,8	21,6	22,6	23,3
80	12,3	13	14	15,7	17,6	19,6	21,3	22,3	23
85	12	12,7	13,7	15,4	17,4	19,3	21	22	22,7
90	11,7	12,4	13,4	15,1	17	18,9	20,6	21,7	22,3

Supplemental Table 26. Percentiles of paraspinal muscle index (cm2/m2) based on pure-muscle sub-areas in males									
Age (years)	p3	p5	p10	p25	p50	p75	p90	p95	p97
20	11,9	12,6	13,5	15,2	17	18,8	20,4	21,4	22
25	12,5	13,1	14,1	15,8	17,6	19,5	21,1	22,1	22,8
30	12,7	13,4	14,4	16,1	18	19,8	21,5	22,5	23,2
35	12,7	13,4	14,4	16,1	18	19,9	21,6	22,7	23,3
40	12,6	13,3	14,3	16	18	19,9	21,6	22,7	23,3
45	12,4	13,1	14,1	15,9	17,8	19,8	21,5	22,5	23,2
50	12,2	12,9	13,9	15,7	17,6	19,6	21,4	22,4	23,1
55	12	12,7	13,8	15,5	17,5	19,5	21,3	22,3	23
60	11,8	12,5	13,5	15,3	17,3	19,3	21,1	22,1	22,8
65	11,4	12,1	13,2	15	16,9	18,9	20,7	21,8	22,5
70	10,9	11,6	12,7	14,5	16,4	18,4	20,2	21,3	21,9
75	10,3	11	12	13,8	15,8	17,8	19,5	20,6	21,3
80	9,6	10,3	11,4	13,1	15,1	17,1	18,8	19,9	20,5
85	9	9,7	10,7	12,5	14,4	16,4	18,1	19,2	19,9
90	8,4	9,1	10,1	11,9	13,8	15,7	17,5	18,5	19,2

**Supplemental Table 27. Percentiles of paraspinal muscle index (cm2/m2) based on complete muscle areas in females**									
Age (years)	p3	p5	p10	p25	p50	p75	p90	p95	p97
20	10,8	11,3	12	13,3	14,7	16,1	17,3	18,1	18,6
25	10,9	11,4	12,2	13,5	14,9	16,3	17,6	18,4	18,9
30	11	11,5	12,3	13,6	15,1	16,6	17,9	18,7	19,2
35	11,1	11,6	12,5	13,8	15,3	16,9	18,2	19	19,6
40	11,2	11,7	12,6	14	15,6	17,1	18,5	19,4	19,9
45	11,3	11,9	12,7	14,2	15,8	17,4	18,8	19,7	20,3
50	11,4	12	12,8	14,3	16	17,7	19,1	20	20,6
55	11,5	12,1	13	14,5	16,2	17,9	19,5	20,4	21
60	11,5	12,1	13,1	14,7	16,4	18,2	19,8	20,7	21,3
65	11,6	12,2	13,2	14,8	16,6	18,5	20,1	21,1	21,7
70	11,7	12,3	13,3	15	16,9	18,7	20,4	21,4	22,1
75	11,7	12,4	13,4	15,2	17,1	19	20,7	21,8	22,4
80	11,8	12,5	13,6	15,3	17,3	19,3	21,1	22,1	22,8
85	11,9	12,6	13,7	15,5	17,5	19,6	21,4	22,5	23,2
90	11,9	12,6	13,8	15,6	17,7	19,8	21,7	22,8	23,6

**Supplemental Table 28. Percentiles of paraspinal muscle index (cm2/m2) based on pure-muscle sub-areas in females**									
Age (years)	p3	p5	p10	p25	p50	p75	p90	p95	p97
20	10,7	11,2	11,9	13,1	14,4	15,7	16,9	17,6	18
25	10,8	11,3	12	13,2	14,5	15,9	17,1	17,8	18,3
30	10,9	11,4	12,1	13,3	14,7	16,1	17,3	18	18,5
35	11	11,4	12,2	13,5	14,8	16,2	17,5	18,2	18,7
40	11	11,5	12,3	13,5	14,9	16,4	17,6	18,4	18,9
45	11	11,5	12,2	13,6	15	16,4	17,7	18,5	19
50	10,9	11,4	12,2	13,5	15	16,5	17,8	18,6	19,1
55	10,7	11,3	12,1	13,4	14,9	16,4	17,8	18,6	19,1
60	10,5	11	11,9	13,3	14,8	16,3	17,7	18,5	19,1
65	10,2	10,8	11,6	13	14,6	16,2	17,6	18,4	19
70	9,9	10,5	11,3	12,8	14,4	16	17,4	18,2	18,8
75	9,5	10,1	11	12,5	14,1	15,7	17,2	18,1	18,6
80	9,2	9,7	10,6	12,1	13,8	15,4	16,9	17,8	18,4
85	8,7	9,3	10,2	11,8	13,5	15,2	16,7	17,6	18,2
90	8,3	8,9	9,8	11,4	13,1	14,8	16,4	17,3	17,9

Supplemental Table 29. Percentiles of paraspinal muscle attenuation (Hounsfield units) based on complete muscle areas in males									
Age (years)	p3	p5	p10	p25	p50	p75	p90	p95	p97
20	46,6	48,4	51,2	55,9	61,1	66,3	71	73,8	75,6
25	43,9	45,9	48,9	54	59,6	65,3	70,4	73,4	75,4
30	40,9	43	46,3	51,8	57,9	64,1	69,6	72,9	75
35	37,3	39,6	43,2	49,1	55,8	62,4	68,4	71,9	74,3
40	32,9	35,5	39,3	45,8	53	60,2	66,6	70,5	73
45	28	30,8	35	42	49,8	57,6	64,6	68,8	71,5
50	22,7	25,6	30,2	37,8	46,2	54,7	62,3	66,8	69,8
55	17	20,2	25,1	33,3	42,5	51,7	59,9	64,8	68
60	10,9	14,4	19,8	28,7	38,6	48,5	57,5	62,8	66,3
65	4,6	8,3	14,1	23,8	34,6	45,3	55	60,8	64,6
70	-2,2	1,8	8,1	18,6	30,3	42	52,5	58,7	62,8
75	-9,6	-5,2	1,7	13	25,7	38,3	49,7	56,5	61
80	-17,3	-12,5	-5,2	7,2	20,9	34,6	47	54,3	59,1
85	-25,4	-20,2	-12,2	1,2	16,1	30,9	44,3	52,3	57,5
90	-33,6	-28	-19,3	-4,8	11,3	27,4	41,9	50,6	56,2

Supplemental Table 30. Percentiles of paraspinal muscle attenuation (Hounsfield units) based on pure-muscle sub-areas in males									
Age (years)	p3	p5	p10	p25	p50	p75	p90	p95	p97
20	49	50,6	53,2	57,5	62,2	67	71,3	73,9	75,5
25	46,8	48,6	51,3	55,9	61	66	70,6	73,3	75,1
30	44,4	46,3	49,2	54,1	59,5	64,9	69,8	72,7	74,6
35	41,6	43,6	46,7	51,9	57,7	63,4	68,6	71,7	73,7
40	38,4	40,5	43,8	49,3	55,4	61,5	67,1	70,3	72,5
45	34,8	37,1	40,6	46,4	52,9	59,4	65,2	68,7	71
50	31,1	33,5	37,2	43,3	50,2	57	63,2	66,9	69,3
55	27,3	29,8	33,7	40,2	47,4	54,6	61,1	65	67,5
60	23,5	26,1	30,2	37	44,6	52,1	58,9	63	65,6
65	19,8	22,5	26,7	33,8	41,7	49,5	56,6	60,8	63,6
70	16,1	18,9	23,3	30,6	38,7	46,8	54,1	58,4	61,3
75	12,4	15,3	19,8	27,2	35,5	43,9	51,3	55,8	58,7
80	8,7	11,6	16,2	23,8	32,3	40,8	48,4	53	56
85	5	8	12,7	20,5	29,1	37,7	45,5	50,2	53,2
90	1,4	4,4	9,2	17,1	25,9	34,7	42,6	47,3	50,4

Supplemental Table 31. Percentiles of paraspinal muscle attenuation (Hounsfield units) based on complete areas in females									
Age (years)	p3	p5	p10	p25	p50	p75	p90	p95	p97
20	42,3	44,3	47,3	52,3	57,8	63,4	68,4	71,4	73,4
25	39	41,1	44,3	49,8	55,8	61,8	67,3	70,5	72,6
30	35,2	37,5	41	46,9	53,4	59,9	65,8	69,4	71,6
35	30,9	33,3	37,1	43,5	50,6	57,6	64	67,8	70,3
40	26,2	28,8	32,9	39,8	47,4	55	61,9	66	68,7
45	21,2	24,1	28,5	35,9	44,1	52,3	59,7	64,1	67
50	15,9	19	23,7	31,7	40,5	49,3	57,2	62	65
55	10,1	13,4	18,5	26,9	36,4	45,8	54,3	59,4	62,7
60	3,8	7,3	12,7	21,7	31,7	41,7	50,7	56,1	59,6
65	-2,7	1	6,6	16,1	26,6	37	46,5	52,1	55,8
70	-9,2	-5,4	0,4	10,1	21	31,8	41,5	47,3	51,1
75	-15,5	-11,7	-5,8	4,1	15	26	35,9	41,8	45,6
80	-21,6	-17,8	-11,9	-2,1	8,8	19,8	29,6	35,5	39,3
85	-27,5	-23,8	-18	-8,3	2,5	13,2	22,9	28,7	32,5
90	-33,4	-29,7	-24	-14,5	-4	6,5	16	21,7	25,4

Supplemental Table 32. Percentiles of paraspinal muscle attenuation (Hounsfield units) based on pure-muscle sub-areas in females									
Age (years)	p3	p5	p10	p25	p50	p75	p90	p95	p97
20	46,9	48,6	51,1	55,4	60,1	64,8	69,1	71,6	73,3
25	44,5	46,3	49	53,5	58,5	63,4	67,9	70,6	72,4
30	41,8	43,7	46,5	51,3	56,5	61,8	66,5	69,4	71,2
35	38,8	40,7	43,7	48,7	54,3	59,8	64,8	67,8	69,8
40	35,6	37,6	40,8	46	51,9	57,7	63	66,1	68,1
45	32,3	34,4	37,7	43,2	49,4	55,5	61	64,3	66,5
50	28,8	31	34,5	40,3	46,7	53,2	59	62,4	64,7
55	25	27,4	31	37,1	43,8	50,6	56,6	60,3	62,6
60	20,9	23,4	27,2	33,5	40,5	47,6	53,9	57,7	60,2
65	16,6	19,2	23,1	29,7	37	44,3	50,8	54,7	57,3
70	12,3	14,9	19	25,7	33,1	40,6	47,3	51,4	54
75	8	10,7	14,8	21,5	29,1	36,6	43,4	47,5	50,1
80	3,8	6,5	10,5	17,3	24,8	32,4	39,1	43,2	45,8
85	-0,3	2,3	6,3	13	20,4	27,9	34,6	38,6	41,2
90	-4,4	-1,9	2,1	8,7	16	23,3	29,9	33,9	36,4

Supplemental Table 33. Percentiles of visceral fat area (cm2) in males									
Age (years)	p3	p5	p10	p25	p50	p75	p90	p95	p97
20	5,8	7,8	11,6	20,9	36,5	58,6	84,6	103,1	116,4
25	8,3	11	16,4	29,3	50,8	81	116,6	141,9	160,1
30	11,1	14,6	21,5	38,2	65,6	104,1	149,3	181,4	204,3
35	14	18,3	26,9	47,1	80,3	126,5	180,6	219	246,4
40	17,5	22,7	33	57,3	96,7	151,2	214,8	259,8	292
45	21,6	27,9	40,1	68,7	114,8	178,3	252	304,1	341,2
50	26,1	33,5	47,7	80,7	133,4	205,4	288,7	347,4	389,3
55	30,8	39,2	55,3	92,4	151	230,6	322,2	386,7	432,5
60	35,2	44,4	62,1	102,1	164,8	249,5	346,4	414,3	462,6
65	38,5	48,2	66,5	107,7	171,6	257,1	354,5	422,5	470,8
70	40,7	50,5	68,9	109,7	172,2	255,2	349,1	414,5	460,8
75	43,3	53,2	71,7	112,1	173,3	253,8	344,3	407,1	451,5
80	46,9	57,1	75,9	116,6	177,4	256,5	345	406,1	449,2
85	50,9	61,4	80,5	121,6	182,1	260,2	346,9	406,5	448,5
90	54,9	65,7	85,1	126,2	186,3	263	347,6	405,6	446,4

Supplemental Table 34. Percentiles of visceral fat area (cm2) in females									
Age (years)	p3	p5	p10	p25	p50	p75	p90	p95	p97
20	7,5	9,2	12,3	19	29,2	42,6	57,5	67,9	75,2
25	7,7	9,6	13,2	21,2	33,7	50,3	69,3	82,5	91,8
30	7,9	10	14,2	23,6	38,6	58,9	82,3	98,8	110,5
35	8,2	10,6	15,3	26,2	43,9	68,3	96,7	116,7	131
40	8,6	11,3	16,6	29,2	49,8	78,7	112,5	136,5	153,7
45	9,2	12,2	18,2	32,6	56,5	90,2	129,9	158,1	178,3
50	10,3	13,6	20,4	36,7	64	102,5	147,9	180,3	203,5
55	11,9	15,7	23,3	41,8	72,3	115,4	166	202,1	227,9
60	14,2	18,6	27,2	47,9	81,7	128,9	184,1	223,3	251,3
65	17,2	22,2	31,9	54,6	91,1	141,4	199,7	240,9	270,3
70	20,7	26,2	36,7	60,7	98,5	149,7	208,4	249,6	278,9
75	24	29,8	40,7	65	102,4	152	208,2	247,4	275,2
80	26,5	32,4	43,3	66,9	102,4	148,9	200,8	236,8	262,2
85	27,9	33,7	44,1	66,4	99,3	141,7	188,7	221,1	243,9
90	28,4	33,8	43,6	64,3	94,3	132,5	174,5	203,2	223,4

Supplemental Table 35. Percentiles of visceral fat attenuation (Hounsfield units) in males									
Age (years)	p3	p5	p10	p25	p50	p75	p90	p95	p97
20	-98,3	-91,7	-81,5	-64,4	-45,5	-26,6	-9,5	0,7	7,3
25	-105,9	-99,5	-89,7	-73,3	-55	-36,8	-20,4	-10,6	-4,2
30	-110,8	-104,6	-95,2	-79,4	-61,8	-44,2	-28,4	-18,9	-12,8
35	-114,2	-108,2	-99,1	-83,9	-66,9	-50	-34,8	-25,7	-19,7
40	-117,4	-111,7	-102,9	-88,2	-71,9	-55,6	-40,9	-32,2	-26,4
45	-120,2	-114,7	-106,2	-92,1	-76,4	-60,6	-46,5	-38	-32,5
50	-121,7	-116,4	-108,2	-94,6	-79,4	-64,3	-50,7	-42,5	-37,2
55	-121,8	-116,7	-108,8	-95,7	-81,1	-66,5	-53,4	-45,5	-40,4
60	-120,4	-115,4	-107,9	-95,2	-81,2	-67,1	-54,5	-46,9	-42
65	-117,6	-112,8	-105,5	-93,4	-79,8	-66,3	-54,1	-46,8	-42
70	-114,8	-110,2	-103,2	-91,4	-78,4	-65,3	-53,6	-46,6	-42
75	-113,8	-109,4	-102,7	-91,3	-78,8	-66,2	-54,9	-48,1	-43,7
80	-114	-109,7	-103,2	-92,3	-80,2	-68,1	-57,2	-50,6	-46,4
85	-113,2	-109,1	-102,9	-92,4	-80,7	-69	-58,5	-52,2	-48,1
90	-111,2	-107,2	-101,2	-91	-79,8	-68,6	-58,4	-52,4	-48,4

Supplemental Table 36. Percentiles of visceral fat attenuation (Hounsfield units) in females									
Age (years)	p3	p5	p10	p25	p50	p75	p90	p95	p97
20	-88,7	-82,7	-73,5	-58,1	-41	-24	-8,6	0,6	6,6
25	-93	-86,9	-77,5	-61,8	-44,4	-27	-11,3	-1,9	4,2
30	-97,2	-91	-81,5	-65,5	-47,8	-30,2	-14,2	-4,7	1,5
35	-101,1	-94,9	-85,3	-69,2	-51,3	-33,4	-17,4	-7,7	-1,5
40	-104,7	-98,5	-88,8	-72,7	-54,7	-36,8	-20,7	-11	-4,7
45	-107,9	-101,7	-92	-76	-58,1	-40,2	-24,1	-14,5	-8,3
50	-110,6	-104,5	-94,9	-79	-61,4	-43,7	-27,8	-18,3	-12,1
55	-112,9	-106,8	-97,5	-81,9	-64,6	-47,3	-31,7	-22,4	-16,3
60	-114,6	-108,7	-99,7	-84,5	-67,7	-51	-35,8	-26,8	-20,9
65	-115,5	-109,9	-101,2	-86,7	-70,6	-54,5	-40	-31,3	-25,7
70	-115,5	-110,2	-101,9	-88,1	-72,8	-57,5	-43,7	-35,4	-30,1
75	-114,6	-109,5	-101,7	-88,6	-74,1	-59,5	-46,4	-38,6	-33,5
80	-112,9	-108	-100,6	-88,1	-74,3	-60,5	-48	-40,6	-35,8
85	-110,6	-106	-98,9	-86,9	-73,7	-60,5	-48,6	-41,4	-36,8
90	-108	-103,6	-96,7	-85,3	-72,6	-59,9	-48,4	-41,6	-37,1

Supplemental Table 37. Percentiles of subcutaneous fat area (cm2) in males									
Age (years)	p3	p5	p10	p25	p50	p75	p90	p95	p97
20	12,5	17,1	26,7	51,3	93,9	156	230,7	284,5	323,2
25	16	21,3	32,2	58,9	103,8	167,7	243,4	297,5	336,3
30	19,9	26	38	66,5	113,1	178	253,9	307,7	346,1
35	24,2	30,9	43,8	73,8	121,5	186,7	261,9	314,8	352,5
40	28,7	36	49,9	81	129,4	194,3	268,2	319,9	356,6
45	33,6	41,3	55,9	87,9	136,4	200,4	272,5	322,6	358,1
50	38,4	46,6	61,6	93,9	142,1	204,6	274,2	322,3	356,1
55	42,9	51,3	66,5	98,7	145,6	205,8	272	317,5	349,4
60	46,5	54,9	69,9	101,2	146,1	202,7	264,4	306,6	336,1
65	49,1	57,3	71,8	101,5	143,4	195,6	252,1	290,4	317,1
70	50,9	58,8	72,5	100,3	139	186,5	237,5	271,9	295,9
75	52,3	59,8	72,8	98,7	134,3	177,5	223,5	254,3	275,7
80	53,5	60,6	72,8	96,9	129,5	168,8	210,3	237,9	257
85	54,1	60,8	72,1	94,4	124,1	159,6	196,7	221,3	238,3
90	54,1	60,3	70,8	91,2	118,1	149,9	182,9	204,7	219,8

Supplemental Table 38. Percentiles of subcutaneous fat area (cm2) in females									
Age (years)	p3	p5	p10	p25	p50	p75	p90	p95	p97
20	32,7	41,4	58,2	96,7	157,3	239,4	333,8	400	447,1
25	35,1	44,4	62	102,1	164,9	249,8	346,9	415	463,4
30	37,8	47,5	65,9	107,6	172,5	259,7	359,2	428,9	478,4
35	40,6	50,7	69,9	112,9	179,6	268,7	370	440,8	491
40	43,4	53,9	73,8	118	185,8	276,1	378,3	449,6	500,1
45	46,3	57,1	77,5	122,4	190,8	281,3	383,3	454,3	504,5
50	49,1	60,3	80,9	126,1	194,3	283,8	384,2	453,9	503,1
55	52	63,3	84,1	129	196,1	283,4	380,9	448,3	495,9
60	55,1	66,5	87,1	131,4	196,8	281	374,5	438,9	484,2
65	58,5	69,8	90,3	133,6	196,7	277,2	365,8	426,6	469,3
70	62	73,2	93,3	135,3	195,6	271,7	354,8	411,5	451,2
75	65,1	76,1	95,7	136	193	264,2	341,4	393,8	430,4
80	67,3	78	96,8	135,1	188,5	254,6	325,7	373,7	407,2
85	68,4	78,6	96,5	132,4	182	242,8	307,7	351,4	381,8
90	68,6	78,3	95	128,5	174,2	229,8	288,8	328,3	355,7

Supplemental Table 39. Percentiles of subcutaneous fat attenuation (Hounsfield units) in males									
Age (years)	p3	p5	p10	p25	p50	p75	p90	p95	p97
20	-119,2	-113,9	-105,6	-91,9	-76,6	-61,3	-47,6	-39,3	-34
25	-119,9	-114,9	-107,2	-94,2	-79,9	-65,5	-52,5	-44,8	-39,8
30	-120,4	-115,6	-108,3	-96,2	-82,6	-69,1	-56,9	-49,6	-44,9
35	-120,5	-116,1	-109,2	-97,7	-84,9	-72,1	-60,6	-53,7	-49,3
40	-120,6	-116,4	-109,9	-99	-86,8	-74,7	-63,8	-57,3	-53
45	-120,7	-116,6	-110,4	-100	-88,4	-76,8	-66,4	-60,1	-56,1
50	-120,5	-116,6	-110,6	-100,6	-89,4	-78,3	-68,3	-62,3	-58,4
55	-120,1	-116,3	-110,5	-100,7	-89,9	-79,1	-69,3	-63,5	-59,7
60	-119,3	-115,5	-109,8	-100,2	-89,6	-79	-69,4	-63,7	-60
65	-118,1	-114,5	-108,8	-99,4	-88,9	-78,4	-69	-63,3	-59,6
70	-117,1	-113,4	-107,9	-98,5	-88,1	-77,7	-68,4	-62,8	-59,1
75	-116,3	-112,7	-107,1	-97,8	-87,5	-77,2	-67,9	-62,4	-58,8
80	-115,6	-112	-106,5	-97,3	-87	-76,8	-67,5	-62	-58,4
85	-114,8	-111,2	-105,7	-96,6	-86,3	-76,1	-66,9	-61,4	-57,9
90	-113,9	-110,3	-104,8	-95,7	-85,5	-75,3	-66,1	-60,6	-57,1

Supplemental Table 40. Percentiles of subcutaneous fat attenuation (Hounsfield units) in females									
Age (years)	p3	p5	p10	p25	p50	p75	p90	p95	p97
20	-114,6	-111,8	-107,5	-100,3	-92,3	-84,3	-77,1	-72,8	-70
25	-115,6	-112,7	-108,3	-100,9	-92,7	-84,4	-77	-72,6	-69,7
30	-116,8	-113,8	-109,2	-101,6	-93,1	-84,6	-77	-72,4	-69,4
35	-118	-114,9	-110,2	-102,3	-93,6	-84,8	-77	-72,3	-69,2
40	-119,2	-116,1	-111,2	-103,1	-94,1	-85,1	-77	-72,1	-68,9
45	-120,5	-117,2	-112,2	-103,8	-94,6	-85,3	-76,9	-71,9	-68,6
50	-121,7	-118,3	-113,2	-104,5	-95	-85,4	-76,7	-71,6	-68,2
55	-122,8	-119,3	-114	-105,1	-95,2	-85,4	-76,5	-71,2	-67,7
60	-123,8	-120,2	-114,8	-105,6	-95,4	-85,2	-76,1	-70,6	-67
65	-124,7	-121	-115,4	-105,9	-95,4	-84,9	-75,5	-69,8	-66,2
70	-125,5	-121,7	-115,9	-106,1	-95,3	-84,5	-74,8	-68,9	-65,1
75	-126,2	-122,3	-116,2	-106,2	-95,1	-83,9	-73,9	-67,8	-63,9
80	-126,7	-122,7	-116,5	-106,1	-94,6	-83,1	-72,8	-66,6	-62,6
85	-127,1	-122,9	-116,6	-105,9	-94	-82,2	-71,5	-65,1	-61
90	-127,4	-123,2	-116,6	-105,6	-93,4	-81,2	-70,2	-63,6	-59,3

Supplemental Table 41. Percentiles of total fat area (cm2) in males									
Age (years)	p3	p5	p10	p25	p50	p75	p90	p95	p97
20	20,6	27,5	41,4	75,3	132,4	213,4	309,2	377,6	426,7
25	27	35,4	51,9	91,5	156,4	247,1	353,2	428,6	482,5
30	34,4	44,3	63,6	108,6	180,8	280,2	395,3	476,6	534,6
35	42,7	54,1	76	126,1	204,9	311,8	434,4	520,6	581,8
40	52,1	65,1	89,7	144,8	230,1	344	473,5	564	628,2
45	62,7	77,3	104,5	164,6	255,8	376,2	511,9	606,1	672,8
50	74	90,1	119,8	184,3	280,6	406,2	546,4	643,4	711,9
55	85,3	102,7	134,4	202,2	302	430,5	573	671	740
60	95,1	113,4	146,2	215,4	316	444	584,8	681,2	748,9
65	102,5	121	153,9	222,4	320,5	444,3	579,3	671,3	735,8
70	107,8	126	158,2	224,6	318,4	435,6	562,4	648,5	708,7
75	112,7	130,6	162,1	226,2	315,7	426,4	545,5	626	682,1
80	118	135,7	166,5	228,6	314,4	419,6	531,9	607,5	660,1
85	123	140,4	170,5	230,5	312,7	412,4	518,3	589,3	638,5
90	127,4	144,4	173,7	231,4	309,7	404	503,5	569,9	615,9

Supplemental Table 42. Percentiles of total fat area (cm2) in females									
Age (years)	p3	p5	p10	p25	p50	p75	p90	p95	p97
20	42,8	53,4	73,3	117,8	186,4	277,9	381,8	454,3	505,6
25	45,6	56,8	78,1	125,8	199,4	297,7	409,3	487,2	542,4
30	48,7	60,7	83,4	134,3	212,8	317,5	436,4	519,5	578,4
35	52,2	65	89,1	143,1	226,3	337	462,8	550,5	612,6
40	56,1	69,7	95,2	152,2	239,5	355,7	487,3	579	644
45	60,5	74,9	101,7	161,3	252,2	372,6	508,7	603,3	670,3
50	65,6	80,7	108,8	170,5	264	387	525,4	621,4	689,3
55	71,7	87,5	116,5	179,8	274,5	398,2	536,6	632,3	699,8
60	79	95,4	125,3	189,6	284,5	407,1	543,2	637	703,1
65	87,6	104,5	135	199,6	293,4	413,2	545	635,3	698,7
70	96,5	113,7	144,4	208,2	299,3	414,1	539,2	624,4	684,1
75	104,4	121,5	151,7	213,5	300,5	408,6	525,2	604,3	659,4
80	109,4	126,1	155,3	214,2	296	396,4	503,9	576,4	626,8
85	111,1	127,1	154,7	210	285,9	378,3	476,5	542,4	588,2
90	110,3	125,3	151,3	202,6	272,4	356,8	445,9	505,6	546,9

Supplemental Table 43. Percentiles of visceral to subcutaneous fat ratio in males									
Age (years)	p3	p5	p10	p25	p50	p75	p90	p95	p97
20	0,2	0,2	0,2	0,3	0,4	0,6	0,7	0,8	0,9
25	0,2	0,2	0,3	0,4	0,5	0,7	0,9	1	1,1
30	0,2	0,2	0,3	0,4	0,6	0,8	1	1,1	1,2
35	0,2	0,3	0,3	0,5	0,7	0,9	1,1	1,3	1,4
40	0,3	0,3	0,4	0,5	0,8	1	1,3	1,5	1,7
45	0,3	0,3	0,4	0,6	0,9	1,2	1,5	1,7	1,9
50	0,3	0,4	0,5	0,7	1	1,3	1,7	2	2,1
55	0,3	0,4	0,5	0,7	1	1,4	1,9	2,2	2,4
60	0,4	0,4	0,6	0,8	1,1	1,6	2	2,3	2,6
65	0,4	0,5	0,6	0,8	1,2	1,7	2,2	2,5	2,7
70	0,4	0,5	0,6	0,9	1,3	1,8	2,3	2,6	2,9
75	0,4	0,5	0,7	0,9	1,3	1,8	2,4	2,8	3
80	0,5	0,6	0,7	1	1,4	1,9	2,5	2,9	3,2
85	0,5	0,6	0,7	1,1	1,5	2	2,6	3	3,3
90	0,5	0,6	0,8	1,1	1,6	2,2	2,8	3,2	3,5

Supplemental Table 44. Percentiles of visceral to subcutaneous fat ratio in females									
Age (years)	p3	p5	p10	p25	p50	p75	p90	p95	p97
20	0,1	0,1	0,1	0,1	0,2	0,3	0,3	0,4	0,4
25	0,1	0,1	0,1	0,2	0,2	0,3	0,3	0,4	0,4
30	0,1	0,1	0,1	0,2	0,2	0,3	0,4	0,4	0,4
35	0,1	0,1	0,1	0,2	0,2	0,3	0,4	0,4	0,5
40	0,1	0,1	0,1	0,2	0,3	0,3	0,4	0,5	0,5
45	0,1	0,1	0,2	0,2	0,3	0,4	0,5	0,5	0,6
50	0,1	0,1	0,2	0,2	0,3	0,4	0,6	0,6	0,7
55	0,1	0,1	0,2	0,3	0,4	0,5	0,7	0,8	0,8
60	0,1	0,2	0,2	0,3	0,4	0,6	0,8	0,9	1
65	0,1	0,2	0,2	0,3	0,5	0,7	0,9	1,1	1,2
70	0,1	0,2	0,2	0,3	0,5	0,7	1	1,2	1,3
75	0,2	0,2	0,2	0,4	0,5	0,8	1	1,2	1,3
80	0,2	0,2	0,3	0,4	0,5	0,8	1	1,1	1,2
85	0,2	0,2	0,3	0,4	0,6	0,7	0,9	1,1	1,2
90	0,2	0,3	0,3	0,4	0,6	0,7	0,9	1	1,1

Supplemental Table 45. Percentiles of fat to muscle ratio in males									
Age (years)	p3	p5	p10	p25	p50	p75	p90	p95	p97
20	0,1	0,2	0,3	0,5	0,8	1,2	1,7	2	2,2
25	0,2	0,2	0,3	0,5	0,9	1,3	1,8	2,2	2,4
30	0,2	0,3	0,4	0,6	1	1,5	2	2,4	2,7
35	0,3	0,3	0,4	0,7	1,1	1,6	2,2	2,6	2,8
40	0,3	0,4	0,5	0,8	1,2	1,7	2,3	2,8	3
45	0,4	0,5	0,6	0,9	1,3	1,9	2,5	2,9	3,2
50	0,4	0,5	0,7	1	1,5	2	2,7	3,1	3,4
55	0,5	0,6	0,8	1,1	1,6	2,2	2,8	3,3	3,6
60	0,6	0,7	0,8	1,2	1,7	2,3	2,9	3,4	3,7
65	0,6	0,7	0,9	1,3	1,7	2,3	3	3,4	3,7
70	0,7	0,8	1	1,3	1,8	2,4	3	3,4	3,7
75	0,7	0,8	1	1,4	1,8	2,4	3	3,4	3,6
80	0,8	0,9	1,1	1,4	1,9	2,4	3	3,4	3,6
85	0,8	0,9	1,1	1,5	1,9	2,4	3	3,4	3,6
90	0,9	1	1,2	1,5	1,9	2,5	3	3,4	3,6

Supplemental Table 46. Percentiles of fat to muscle ratio in females									
Age (years)	p3	p5	p10	p25	p50	p75	p90	p95	p97
20	0,4	0,5	0,6	1	1,5	2,1	2,9	3,4	3,7
25	0,4	0,5	0,7	1	1,5	2,2	3	3,5	3,9
30	0,4	0,5	0,7	1,1	1,6	2,3	3,1	3,7	4,1
35	0,5	0,6	0,8	1,2	1,7	2,5	3,3	3,8	4,2
40	0,5	0,6	0,8	1,2	1,8	2,6	3,4	4	4,4
45	0,6	0,7	0,9	1,3	1,9	2,7	3,5	4,1	4,5
50	0,6	0,7	0,9	1,4	2	2,8	3,6	4,2	4,6
55	0,7	0,8	1	1,5	2,1	2,9	3,7	4,3	4,7
60	0,7	0,9	1,1	1,5	2,2	2,9	3,8	4,3	4,7
65	0,8	0,9	1,2	1,6	2,2	3	3,8	4,4	4,8
70	0,9	1	1,2	1,7	2,3	3	3,8	4,4	4,7
75	0,9	1,1	1,3	1,7	2,3	3	3,8	4,3	4,6
80	1	1,1	1,3	1,7	2,3	2,9	3,6	4,1	4,4
85	0,9	1,1	1,3	1,6	2,2	2,8	3,4	3,8	4,1
90	0,9	1	1,2	1,6	2	2,6	3,1	3,5	3,8
